# SorghumHub: featuring the next phase for the KBCommons web portal with new molecular biology features

**DOI:** 10.1186/s12864-026-13113-y

**Published:** 2026-07-16

**Authors:** Nathan Grant, Yen On Chan, Anser Mahmood, Jana Biová, Mária Škrabišová, Trupti Joshi, Kristin Bilyeu

**Affiliations:** 1 Agricultural Research Service - Midwest Area - Plant Genetics Research Unit, Department of Agriculture-Agricultural Research Service, Columbia, MO USA; 2 Christopher S. Bond Life Sciences Center, University of Missouri-Columbia, Columbia, MO USA; 3 Department of Biomedical Sciences, Joan C. Edwards School of Medicine, Marshall University, Huntington, WV USA; 4 Division of Plant Science and Technology, University of Missouri-Columbia, Columbia, MO USA; 5 Department of Biochemistry, Faculty of Science, Palacký University in Olomouc, Olomouc, Czechia; 6 MU Institute for Data Science and Informatics, University of Missouri-Columbia, Columbia, MO USA; 7 Department of Biomedical Informatics, Biostatistics, and Medical Epidemiology, School of Medicine, University of Missouri-Columbia, Columbia, MO USA

**Keywords:** SorghumHub, Sorghum Allele Catalog Tool, Protein Sequence Logos, Allele Exploration, Phenotype Improvement, *Sorghum bicolor*, Applied Genomics, Alleles, Whole Genome Resequencing (WGRS)

## Abstract

**Background:**

Genomic resources for underutilized crops like sorghum [*Sorghum bicolor* (L.) Moench] often lag behind major staples, hindering efforts to link genetic diversity to agronomic traits. Improvements in genomic sequencing and bioinformatics have advanced crop genomics, yet species-specific challenges persist in the lack of tailored genomic resources, limiting precision breeding and applied genomics applications.

**Results:**

This study presents the addition of a SorghumHub to the KBCommons web portal, an applied genomics platform for Sorghum. The core focus of this framework is the Sorghum Allele Catalog Tool, with a web-based interface enabling researchers to query, visualize, and download allelic variants from curated sorghum datasets of 988 resequenced accessions. The newly improved Allele Catalog Tool now features the ability to connect variant positions with phenotype information collected for Plant Introduction (PI) accessions in the Germplasm Resource Information Network (GRIN). On the SorghumHub, users can also find the Protein Sequence Logos, a new tool complementary to the Allele Catalog Tool, which allows users to graphically explore missense amino acid sequence changes for conservation and frequency. These custom genomic tools available on the SorghumHub enable users to query genomic variants, allele frequency, and trait-associated genotype–phenotype relationships leveraging data produced through our group’s computational pipelines for variant calling and processing into curated Allele Catalog datasets.

**Conclusions:**

This research work demonstrates practical application of Sorghum Allele Catalog Tool and Protein Sequence Logos by highlighting different candidate alleles for the predicted flowering time (FT-like) gene *SbFT12* (*Sobic.006G047700*) in *Sorghum bicolor*; additionally, we discovered a relatively rare missense allele of the betaine aldehyde dehydrogenase (BADH2) gene that may provide a new avenue for fragrant/scented sorghum. The tools in SorghumHub allow researchers to optimize the accession selection process with efficient trait targeting and bridge the gaps between genomics and breeding. This approach can assist breeders and researchers in the development of resilient and high yielding crop varieties with broader positive outcomes for the agricultural industry and global food security.

**Supplementary Information:**

The online version contains supplementary material available at https://doi.org/10.1186/s12864-026-13113-y.

## Background

*Sorghum bicolor* is the fifth most economically important cereal grain crop in the world known for its three ideotypes: grain, forage, and/or bioenergy (i.e. sweet or cellulosic), according to United States Department of Agriculture - National Agriculture Statistics Services (USDA-NASS; www.nass.usda.gov). Sorghum flowering time directly affects yield, adaptation, and end-use quality; therefore, breeders try to target specific environments to improve harvestability [1]. Due to its environmental plasticity, sorghum is a target of crop improvement to safeguard global food and energy security [2]. Sorghum also has a relatively small genome (2n = 20; ~730 Mb) that makes it an excellent C4 grass model for genomic studies. Sorghum is typically a short-day plant, but photoperiod insensitive varieties have been bred for temperate regions to ensure flowering and grain production [1, 3]. In contrast, bioenergy sorghums retain short day sensitivity, flowering late in long-day conditions to maximize vegetative growth for a higher biomass yield [4]. The prolonged vegetative growth under long days enhances stem sugar (sweet) or total biomass (cellulosic) accumulation through delayed flowering and could be useful for bioenergy production [5]. In contrast, day-length neutrality can offer reliable grain yields in diverse environments [6, 7]. Sorghum leaf and grain fragrance is also a consideration for increasing palatability, though limited options for natural diversity are currently available [8–11].

Current and emerging biotechnological advancements offer promising opportunities for sorghum crop improvement. There are over 47,000 sorghum accessions in the USDA National Plant Germplasm System-Germplasm Resources Information Network (NPGS-GRIN) and most are underutilized (Sorghum and Millet Germplasm Committee minutes March 28, 2022). Next-generation sequencing has become an essential tool for obtaining large amounts of genetic information; whole genome resequencing (WGRS) of diverse sorghum accessions, spanning a wide range of geographic origins, offers an untapped genetic resource [12]. A large amount of publicly available genomics and phenomics data has enabled researchers to explore innovative collecting, processing, analyzing, and visualizing methods to gain insights and transform knowledge into applications that achieve crop improvement. However, accessing and processing these large datasets are increasingly difficult due to the enormous file sizes and high computational costs. These large-scale datasets often require high performance computing resources to process and analyze efficiently. This research work illustrates the scalable workflows and computing environments for data collection, processing, and analysis using tools and applications developed and maintained by our research group. The outputs of this large-scale analysis served through SorghumHub supports researchers by providing access to large genomic datasets directly without the need to collect or process data.

This research work introduces the newly incorporated SorghumHub in the KBCommons web portal where users have the ability to explore precomputed datasets for multiple species using a suite of applied genomic tools [13]. *Sorghum bicolor* WGRS data was collected from various sources. The SnakyVC variant calling pipeline and the Allele Catalog pipeline, developed by our group, were utilized to streamline the processing of WGRS data with USDA supported SCINet high performance computing environment. These pipelines generated the Allele Catalog datasets to power the SorghumHub in the KBCommons web portal [14]. The SorghumHub consists of both the Sorghum Allele Catalog Tool and the Sorghum Protein Sequence Logos tool. The Sorghum Allele Catalog Tool consists of a curated core 988 sorghum Allele Catalog dataset (Sbicolor988v5.1) and two supplemental 400 and 499 sorghum Allele Catalog datasets. The Sorghum Allele Catalog Tool offers all the capabilities found in Allele Catalog Tool for other species. The tool features user-friendly input query boxes for searches, and upon submission, it opens a new webpage displaying the results, making it an accessible and efficient platform for studying allelic diversity. The tool identifies variant positions that have modifying effects on genes through changes to the protein sequence and organizes them into distinct alleles for each gene. These alleles are visualized as a list, highlighting the modifying changes relative to the reference genome annotation. The Allele Catalog Tool organizes each allele by frequency with a summary of categorical information for all accessions. With recent improvements, large phenomics data has been integrated into our database and made available in the phenotype viewer in the Allele Catalog Tool. Users can use the phenotype viewer to connect alleles defined by different variant positions to the phenotypic measurements of accessions and visualize the phenotype distributions. This approach helps researchers identify accessions with unique phenotypic traits and enables targeted studies that may lead to crop improvement. Complementing this tool, we introduce the Sorghum Protein Sequence Logos tool which provides deeper analysis of identified missense mutations and further supports functional exploration of genetic variation.

## Methods

### Datasets

Several sets of available WGRS datasets were collected from various studies in this research. The WGRS datasets included a diverse worldwide Sorghum collection (*n* = 499) and a sorghum association panel (SAP; *n* = 400) [15, 16]. These studies used Illumina short read (2 × 100–150 bp) WGRS technology with sequencing depth averaging 17x and 38x. To expand and create the larger 988 sorghum panel, unique sorghum accessions in FASTQ format from NCBI BioProjects and European Nucleotide Archive (ENA), which include PRJNA74553 (*n* = 3), PRJNA182489 (*n* = 30), PRJNA374837 (*n* = 7), PRJNA513297 (*n* = 214), and PRJEB50066 (*n* = 353), were collected via SRAtools and File Transfer Protocol (FTP). In the curation process, our research group excluded accessions with a threshold of tolerance of less than 10x coverage. The final panel of 988 lines reported here represents a diverse sample set covering the five main sorghum races (bicolor, guinea, caudatum, kafir, and durra) from worldwide locations. Additionally, the open access bioenergy sorghum dataset provided by Dryad’s Transportation Energy Resources from Renewable Agriculture Phenotyping Reference (TERRA-REF) Platform (*n* = 381) was included [12, 15–21].

Apart from WGRS datasets, reference genomes and relevant annotations that are crucial for variant calling and Allele Catalog dataset generation were also collected. The reference genomes include the sorghum reference genome version 3.1.1 (Sbicolor_454_v3.1.1) (https://phytozome-next.jgi.doe.gov/info/Sbicolor_v3_1_1) and Tx623 version 5.1 (Sbicolor.730.v5.1) (https://phytozome-next.jgi.doe.gov/info/Sbicolor_v5_1). Along with these reference genomes, General Feature Format (GFF) annotation files, coding sequences, protein sequences, and transcripts were also collected.

Metadata and Phenotype Data Curation.

The metadata for all samples were collected from the primary resequencing studies [12, 15–21] and phenotype data were curated from the Germplasm Resources Information Network (GRIN) database (https://npgsweb.ars-grin.gov/gringlobal/). The sorghum diversity panel with phenotypes was used to run the Allele Catalog workflow, and to develop an updated Sorghum Allele Catalog Tool [14].

### Variant calling with refined SnakyVC variant calling pipeline

The newly improved SnakyVC variant calling pipeline has incorporated several supplemental workflows to provide more data processing options for large-scale germplasm collections. One of the crucial updates is the option to partition jobs by chromosomes. This divide and conquer approach splits whole genome level data into individual chromosomes during data processing to reduce the computational load per process and allow more efficient utilization of resources and decrease overall runtime. While some of the main workflow steps (i.e., BWA-mem, GATK: SortSam, GATK: MarkDuplicates, GATK: AddOrReplaceReadGroups, and GATK: HaplotypeCaller) scale with the allocated resources, the GATK: CombineGVCFs and GATK: GenotypeGVCFs steps have always remained single threaded. This issue is increasingly inefficient when merging many accessions into a single file. To address this bottleneck, binning by chromosome offers a practical solution by enabling parallelization of processing steps, thereby significantly improving computational efficiency.

In the process of creating sorghum data panel for the Sorghum Allele Catalog Tool, accessions were grouped into batches of 100 and processed using the following three SnakyVC supplemental workflows: (1) BWA_alignment_to_GATK_Haplotype_Caller.smk to streamline analysis from reference based alignment to generate the individual sample Genomic Variant Call Format (GVCF) files and corresponding index files; (2) GATK_CombineGVCFs.smk to combine each batch of accession GVCF files into a multi-accessions GVCF file, and (3) GATK_CombineGVCFs_to GATK_SelectVariants_chromosomewise.smk to merge batched accession GVCF files in parallel, by chromosomes, to generate final sorghum data panel in Variant Call Format (VCF). With these supplemental workflows, variant calling steps are more customizable and simpler to execute by available resources and timeline.

The supplemental workflows were developed, tested, and executed on the USDA supported SCINet high-performance computing infrastructure. These supplemental workflows are publicly available in the ‘workflows’ directory of the SnakyVC GitHub repository (https://github.com/yenon118/snakyVC). Example configuration files for the supplemental workflows can be found in the ‘workflow_inputs’ directory within the same repository.

### Allele frequency based curation and the allele catalog dataset generation

One additional intermediate step required for data curation is to remove positions that contained only heterozygous genotype calls in the VCF files. Any positions that had only heterozygous or homozygous reference, but no alternate homozygous calls recorded were removed to minimize the influence of potential genotyping artifacts, given the predominately inbred nature of sorghum. Frequencies for alternate calls were computed to assess variant representation across samples. Variant positions with an alternate frequency of zero or lacking any reference allele calls were subsequently removed, ensuring that only polymorphic and biologically meaningful positions were retained. This filtering step helped produce a high-confidence variant dataset optimized for downstream analyses. No other filtering was performed so that rare variants would be captured in the curated data.

Upon the completion of data curation, the output sorghum VCF files were inputted into the Allele Catalog pipeline (https://github.com/yenon118/AlleleCatalog) to generate the Allele Catalog datasets. In the Allele Catalog pipeline, any missing genotype data in the VCF files were imputed using Beagle imputation tool; the imputed VCF data was annotated using the SnpEff functional effect prediction tools and aggregated with metadata and GFF files using in-house developed scripts to create the sorghum Allele Catalog datasets. This automated streamline analysis process was completed on the USDA supported SCINet high performance computing cluster.

### Sorghum allele catalog data integration and deployment

To expand the Allele Catalog Tool to support sorghum, the sorghum Allele Catalog datasets were uploaded to a MySQL database integrated with the KBCommons web portal. A dedicated sorghum knowledge base was established within KBCommons to host and manage these datasets and enable structured access and visualization. Following the successful integration and configuration of the database, the Sorghum Allele Catalog Tool was deployed and made publicly accessible through the KBCommons interface. This implementation allows users to query, explore, and analyze allelic variations and phenotype diversity in sorghum across accessions to facilitate genomic research and breeding efforts.

### Sorghum protein sequence logos pipeline and web application

Sorghum Protein Sequence Logos tool outputs protein sequence logos for queried sorghum genes. These logos were generated using the Snakemake based pipeline publicly available at https://github.com/AnserM/PSL/. Briefly, this pipeline was used to conduct BLASTP for protein sequence of all sorghum genes in sorghum reference genome version Tx623 version 5.1 (Sbicolor.730.v5.1) (https://phytozome-next.jgi.doe.gov/info/Sbicolor_v5_1) against NCBI non-redundant database. The BLASTP hits were filtered to retain only the top hit within each genus and the hits with less than 60% sequence similarity were excluded. Additionally, only the top 100 hits, sorted by sequency similarity score, for each query were retained for downstream analysis. The protein sequence for each of the blast hits was extracted from the database and multiple sequence alignments (MSA) were generated using Clustal Omega [22]. The MSA results were visualized using WebLogo [23] where the coordinates of amino acid stacks within the generated logos were anchored to the sorghum query protein sequence coordinates. The Sorghum Protein Sequence Logos were served through an R shiny application hosted at https://www.kbcommons.org/system/tools/ProteinSequenceLogos/Sbicolor. The R shiny application allows users to specify the gene of interest and furnishes the protein sequence logo for the corresponding protein sequence. Additionally, users can specify several gene IDs to download the sequence logos in bulk.

## Results

### SorghumHub: a central hub for sorghum exploration and analytical tools

This research work introduces the newly incorporated SorghumHub in the KBCommons web portal [13]. The SorghumHub currently includes the Sorghum Allele Catalog Tool and the Sorghum Protein Sequence Logos tool (https://kbcommons.org/system/home/Sbicolor/SorghumHub). The Sorghum Allele Catalog Tool consists of our curated data panel of 988 accessions (Sbicolor988v5.1) and two supplemental data panels of 400 and 499 accessions (Sbicolor_454_v3.1.1). Each data panel can be selected based on user preferences in the Sorghum Allele Catalog Tool designed to explore allelic variation and frequency using Gene IDs or accessions [14]. The Sorghum Allele Catalog Tool enhances data accessibility and allows for studying allelic diversity efficiently by featuring user-friendly input query boxes for searches, buttons and checkboxes for data selections, and tables and figures for visualizations and presenting results. The tool identifies gene variant positions that have modifying effects on protein coding regions and organizes them into distinct alleles for each gene. These alleles are visualized as a list, highlighting the modifying changes relative to the reference genome. The Sorghum Allele Catalog Tool groups each allele by the frequency of all accessions found and displays the number of accessions in each preselected category. The upgraded Allele Catalog Tool platform’s enhancements emphasize data connectivity between alleles and phenotypes that now enables phenotype data comparison and analysis at the allele level. To further support the Sorghum Allele Catalog Tool, the Sorghum Protein Sequence Logos tool has been developed and integrated into the SorghumHub for more in-depth analyses of identified missense changes.

## Constructing the Sorghum allele catalog tool and datasets

The original Soybean Allele Catalog Tool is hosted on the SoyKB web portal as part of a SoyHub for soybean applied genomic research and represents a gene-centric platform to explore whole genome sequence variation as it relates to different alleles [14]. With easy expansion capabilities, the Allele Catalog Tool platform has been expanded to support six additional species on the KBCommons space: *Oryza sativa* (rice), *Populus trichocarpa* (Black cottonwood), *Sorghum bicolor*,* Zea mays* (maize), *Arabidopsis thaliana*, and *Phaseolus vulgaris* (common bean) [13]. The Allele Catalog datasets in the new Sorghum Allele Catalog Tool were generated using a reference genome (Sbicolor_v5.1), a General Feature Format (GFF) gene annotation file (Sbicolor_730_v5.1.gene.gff3), and a metadata file (Supplemental Table S1). 

**Fig. 1:**
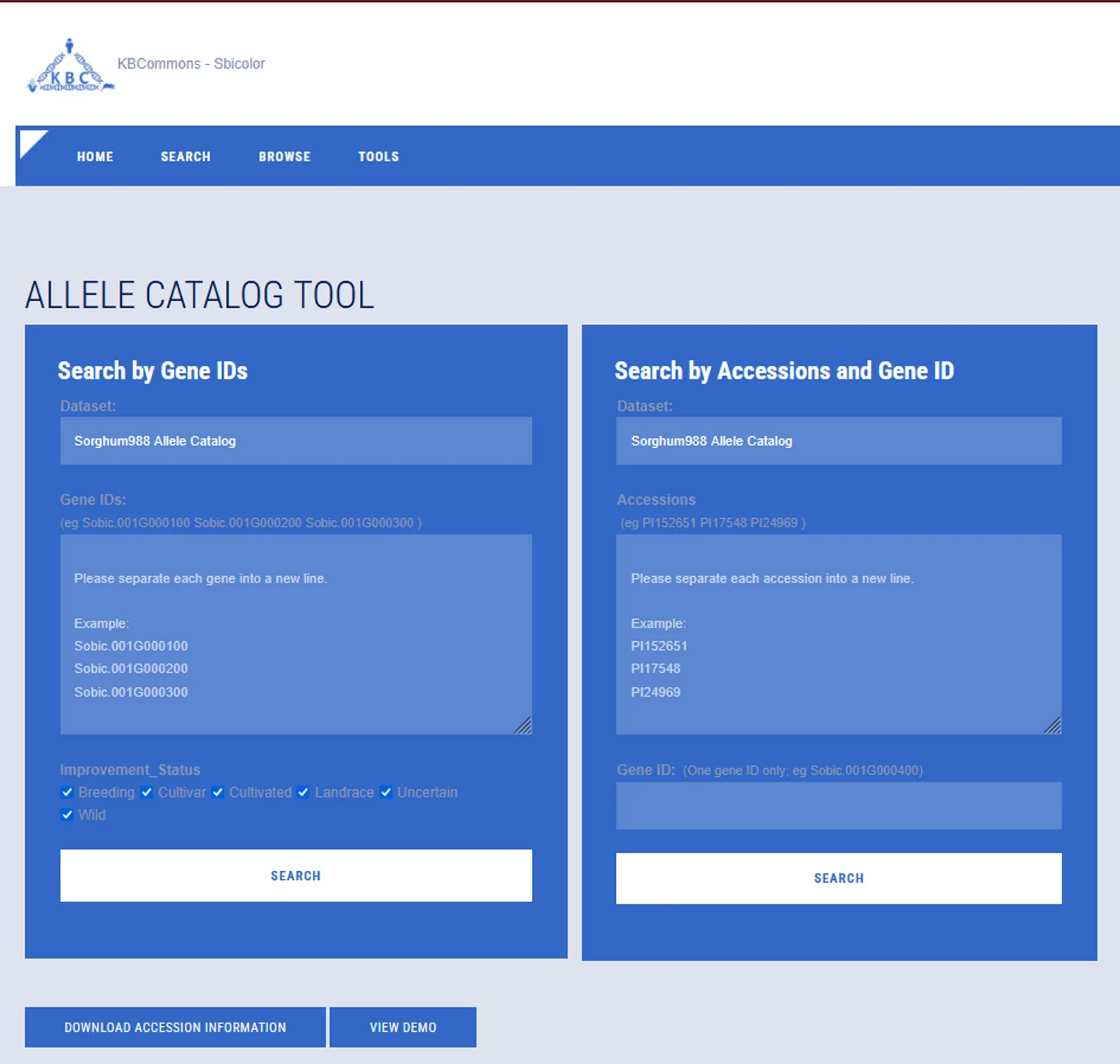
Sorghum Allele Catalog Tool query interface. The web interface facilitates two distinct query pathways for exploring genetic diversity. (Left) The “Search by Gene IDs” function accepts a user-provided list of gene identifiers (e.g. Sobic.001G000100). This returns a comprehensive catalog of all discovered alleles for each gene across the entire sequenced sorghum diversity panel. (Right) The “Search by Accessions and Gene ID” function requires a single gene ID alongside a list of germplasm accessions (e.g. PI 152651). This targeted query returns the specific allele(s) present for that gene within the specified set of accessions, enabling precise genotype-phenotype correlations

**Fig. 2:**
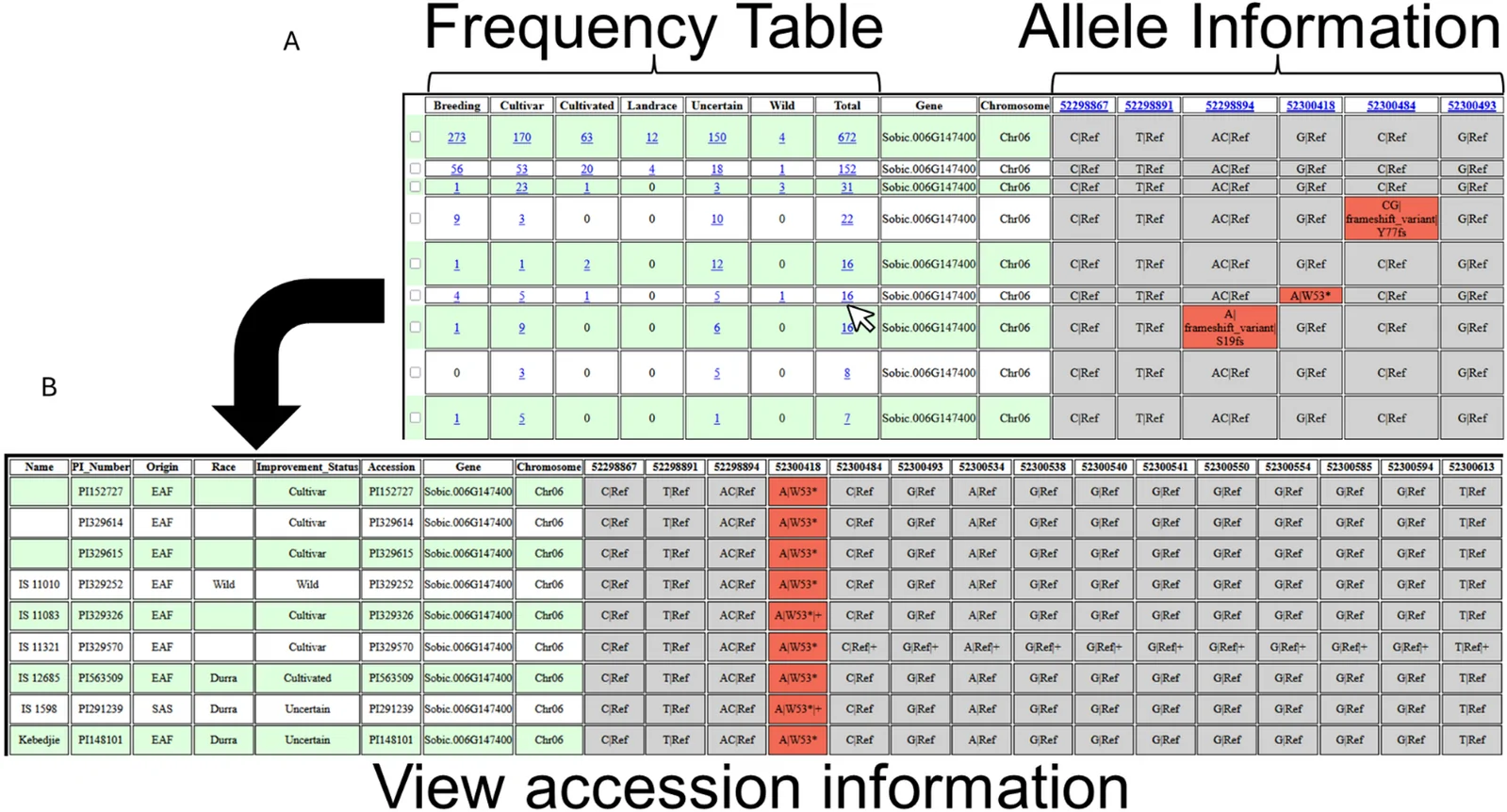
Representative results from a query of the Sorghum Allele Catalog Tool for a single gene (Sobic.006G147400). The output is structured to provide both a population level summary and granular accession details. (**A**) A frequency table lists all discovered alleles for the queried gene. For each allele, the table provides its frequency (count) within the dataset, allowing for rapid assessment of common versus rare variants. (**B**) A detailed accession panel lists the individual plant introduction (PI) lines or accessions that carry each specific allele. This section enables users to link allele-defining variant positions directly to source germplasm, which is critical for downstream applications like association studies or the selection of breeding material

In the Sorghum Allele Catalog Tool on the SorghumHub (Fig. 1), after searching for the gene(s) of interest, categorical variant data is summarized in a results table accompanying each gene’s allele output (Fig. 2A). For the allele information portion of the results allele table, in the heading are each single nucleotide polymorphism (SNP) or insertion or deletion (InDel) variant position. An accompanying frequency results table contains counts of accessions carrying the corresponding alleles in different improvement status subcategories (Fig. 2A). For the Sorghum Allele Catalog, the six subcategories for improvement status based on the GRIN observations are Breeding (*n* = 351), Cultivar (*n* = 292), Cultivated (*n* = 89), Landrace (*n* = 17), Uncertain (*n* = 227), and Wild (*n* = 12).These counts are interactive, allowing users to click on them and be redirected to a popout page with additional details (Fig. 2B). On this page, users can view accessions associated with a specific allele and accession metadata such as Name, PI Number, Origin, and Race. The Sorghum Allele Catalog Tool contains this metadata information to help characterize the details for the various germplasm groups. The heading meanings and totals include Origin that represents the country of origin from GRIN grouped into ten geographic subcategories: WAF: Western Africa (*n* = 158); EAF: Eastern Africa (*n* = 278); NAF: Northern Africa (*n* = 158); SAF: Southern Africa (*n* = 45); SEEU_ME: Southeastern Europe and Middle East (*n* = 13); SAS: South Asia (*n* = 77); EAS: East Asia (*n* = 24); AUS: Australia (*n* = 8); USA: United States (*n* = 150); LAC: Latin America and Caribbean (*n* = 8) and 69 had no GRIN origin associated. Sorghum has five commonly referenced races, and according to the curated GRIN observations, we divided sorghum accession into the following race groups: Bicolor (*n* = 23), Caudatum (*n* = 180), Complex (*n* = 14), Durra (*n* = 176), Guinea (*n* = 180), Kafir (*n* = 58), Wild (*n* = 19); there were 338 accessions that did not have a GRIN observation for Race. Accessions were prioritized based on the plant introduction number (PI) with 922 available from NPGS-GRIN.

Collecting germplasm accessions that have diversity within the genome is essential for identifying useful variants. Variant calling will only genotype predicted differences from a reference genome, highlighting the limitations of using a small germplasm set and inadequate reference genome annotation. For sorghum, version 5.1 is the most recent reference genome for Tx623. Out of the ~ 720 Mb reference genome, our SnakyVC pipeline identified over 55 million total variants among our collection of 988 accessions (Supplemental Table S2). Almost 48 million SNPs and over 6.6 million InDels were predicted. Of the 32,160 predicted genes, 6,375 had no predicted modifying variants while 18,806 genes had at least one predicted null allele in the Sorghum988v5.1 dataset. There are almost 2 million predicted gene modifying variants in the Sorghum Allele Catalog, with an average of 29 alleles per gene (Supplemental Table S2).

###  Genotype and phenotype connection for all predicted allelic variants using GRIN descriptor data with the phenotype data viewing page 

**Fig. 3:**
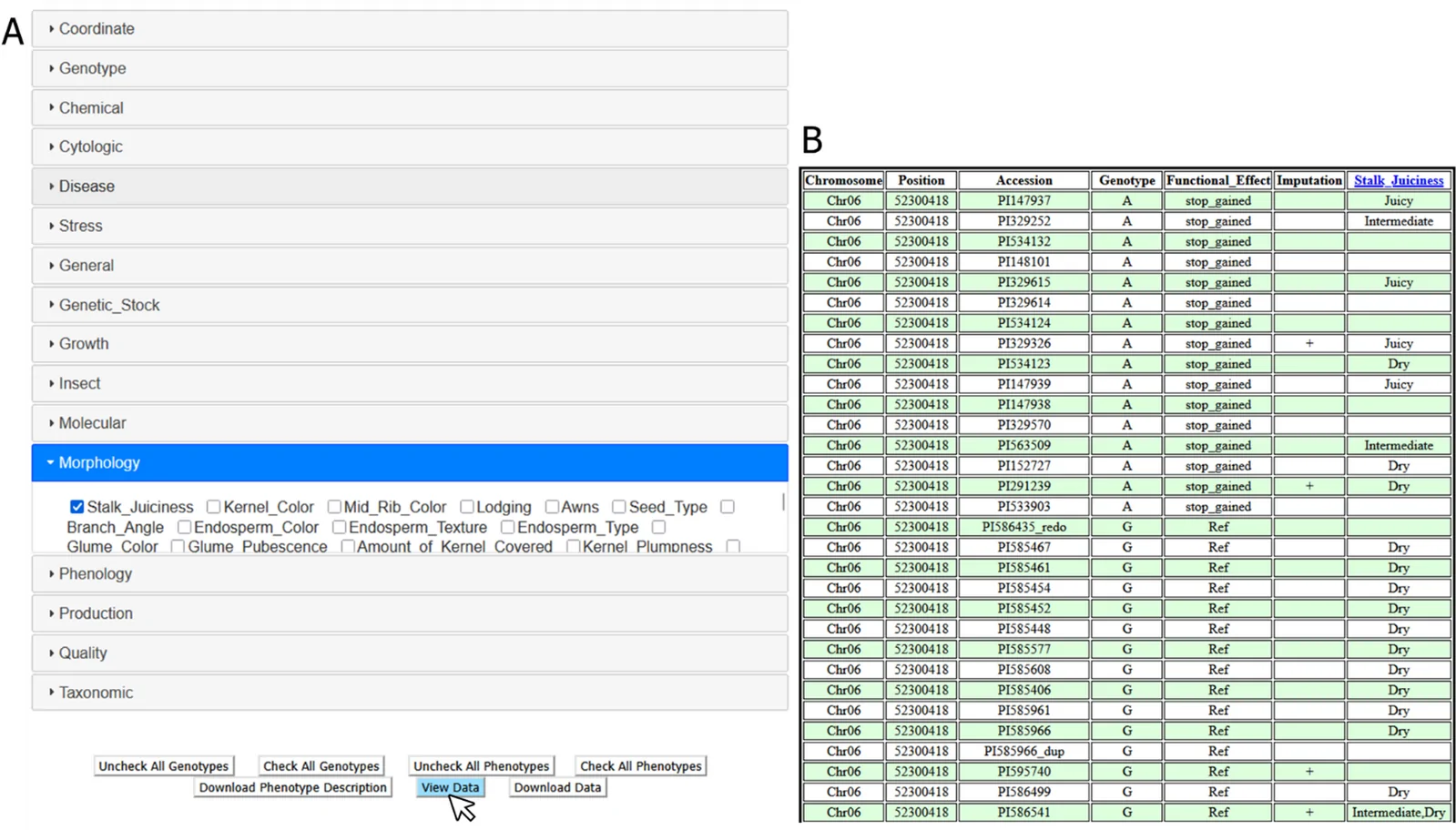
Connecting variants and phenotypes. This interface allows for refined analysis of genotype-phenotype relationships after an initial gene query. The left panel (**A**) displays an accordion style, drop-down distribution of phenotypic values for a chosen trait (e.g. “Stalk Juiciness”) across the accessions carrying the selected variant. Users can select one or multiple phenotype descriptions and view the results in a summary table (**B**). This facilitates visualizing correlations, such as a specific SNP being associated with a higher or lower trait value. Clicking the phenotype descriptor at the top of the summary table links to a graphical representation of the results

Allele mining is a suitable method for dissecting the natural variation among germplasm accessions. When exploring genetic data, it is helpful to make phenotypic comparisons that strengthen evidence for a gene/trait of interest. A new feature on the Allele Catalog Tool main results page is the ability to explore connections between the variant positions and NPGS-GRIN phenotype data based on the framework developed for the GenVarX tool [24]. For situations when the allele’s phenotype effect is not known, the Allele Catalog Tool results table now has a link at each variant position that redirects users to a variant and phenotype data connection page (Fig. 3).

**Fig. 4:**
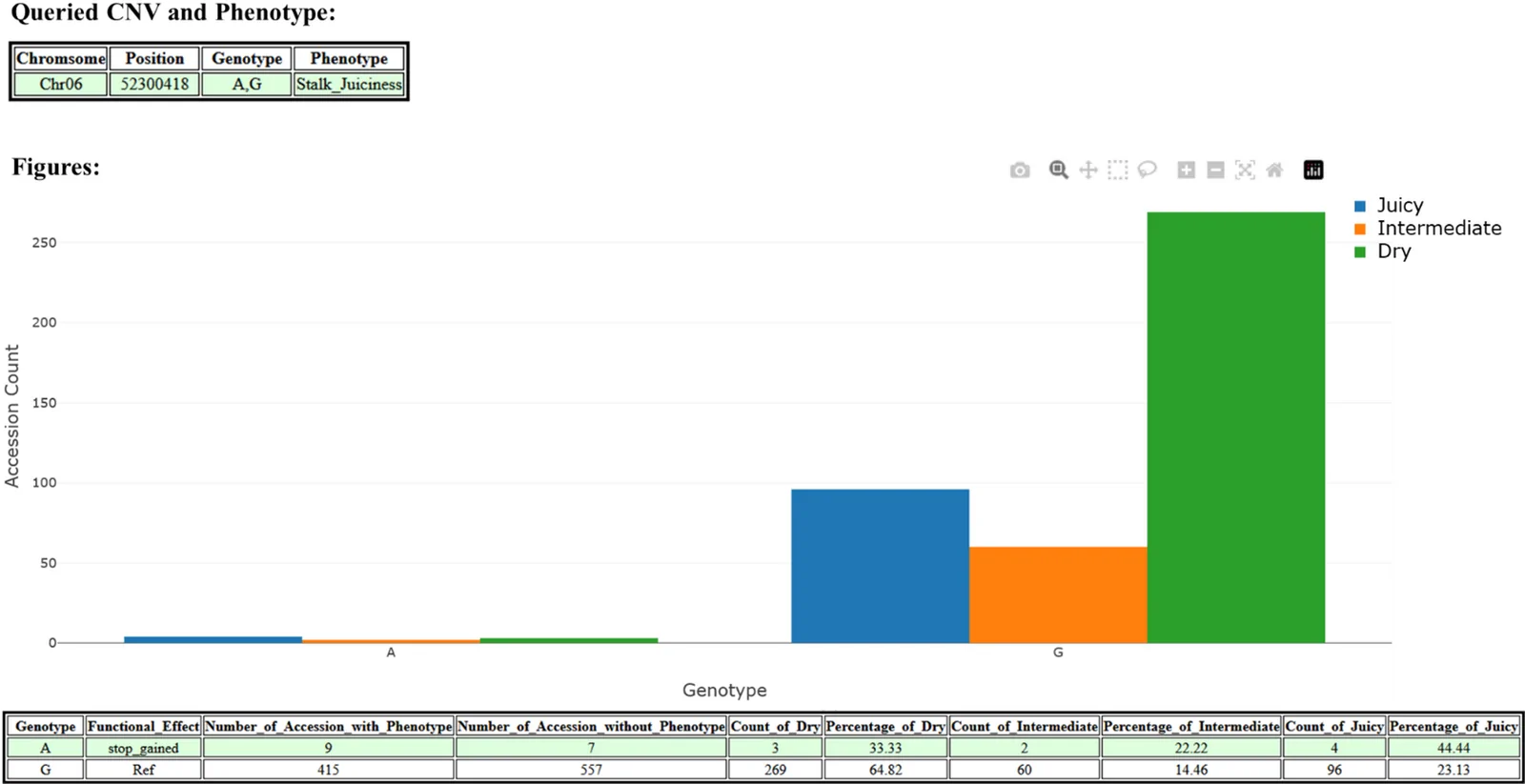
Association between a variant position and a categorical trait. The results visualize the relationship between a queried modifying variant position and a discrete phenotypic outcome. A bar chart showing the proportion of accessions in each phenotypic category (e.g. “Juicy”, “Intermediate” or “Dry” Stalk Juiciness) for different functional effect genotypes (“stop gained” or “reference”). A corresponding contingency table provides the exact numerical counts and percentages used to generate the plot

The phenotype data viewing page features an accordion-style drop-down menu, enabling users to select and view or download phenotype data (Fig. 3). In this example, we explore the key gene controlling stem juiciness, Dry, (*SbNAC_D*; Sobic.006G147400) [25]. Sweet sorghum varieties with juicy stems are used for syrup production and show trade-offs like increased drought tolerance but higher susceptibility to disease and lodging [26, 27]. Now that the gene responsible for stem juiciness is characterized, researchers can use the Sorghum Allele Catalog to rapidly explore allelic variation at this locus and compare it to known phenotypes, streamlining candidate gene analysis without the need for extensive sequencing. To connect genotypes and phenotypes, users use the blue View Data button after selecting/clicking their genotypes and one or more phenotypes (Check All Genotypes and Stalk Juiciness under the Morphology phenotype category as an example; Fig. 3A). A new data tab is opened with the results in table form (Fig. 3B) in which the phenotype headings in the table are clickable. When Stalk Juiciness is selected from this table, another tab is opened with the organized genotype and phenotype results generated as bar plots or violin plots depending on phenotypic variables (Fig. 4). These visualizations help illustrate the distribution of phenotypic data for any selected variant position. Violin plots provide insights into quantitative phenotype data by displaying metrics such as median, minimum, maximum, first quantile, third quantile, and mean. Bar plots reveal the distribution of qualitative or categorical data by displaying counts for each category. These plots help users assess potential relationships and significance between alleles (represented by a variant position) and phenotypes. Below each plot, summary tables provide the user with details on the counts of accessions based on genotypes and either the presence of phenotype data (for quantitative measurements) or the categories (for qualitative measurements). At the bottom of the page, users can also visualize the frequency table distribution across different genotypes, offering further insights into the connections between phenotypes and genotypes (Fig. 4). This comprehensive approach allows users to explore and interpret complex relationships within the data effectively.

### Investigate sequence conservation for missense changes with the sorghum protein sequence logos tool

**Fig. 5:**
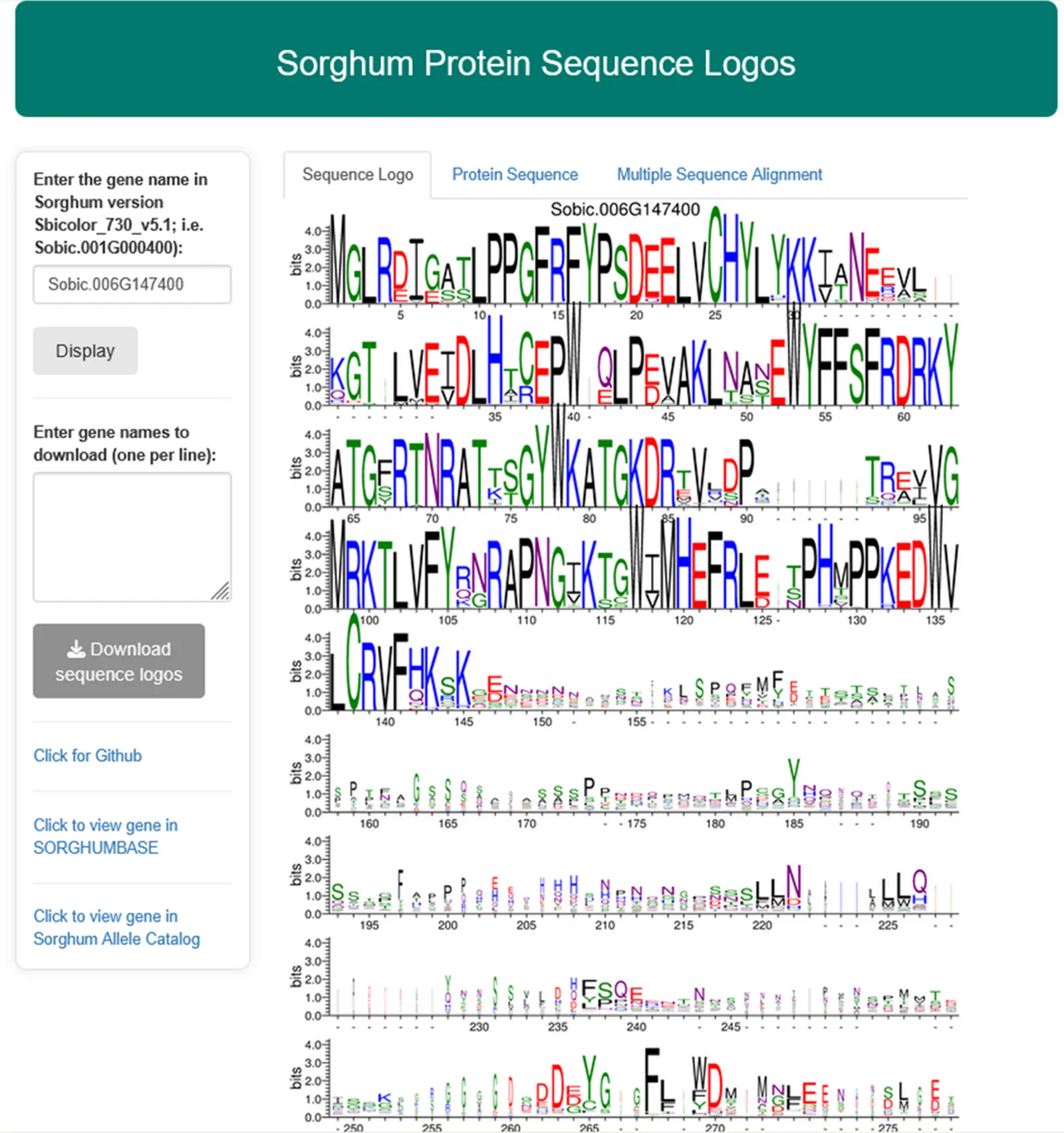
The Sorghum Protein Sequence Logos tool for visualizing conserved protein motifs. The tool’s entry page requires a single sorghum gene identifier (e.g. Sobic.006G147400). Upon submission, the tool gathers the multiple sequence alignment and displays the resulting sequence logo for the protein (Sobic.006G147400). The logo graphically represents amino acid conservation and variation at each amino acid position in the protein sequence. The total height of the stack at each position indicates the sequence conservation (measured in bits of information), while the height of individual letters within the stack represents the relative frequency of each amino acid at that position. This visualization allows for the immediate identification of highly conserved residues (tall stacks with a single letter) critical for protein function, as well as variable regions (short stacks with multiple letters) that may contribute to functional diversity. The tool’s utility is extended by options to download the logo for publication, view individual protein sequence, and examine the multiple sequence alignment directly, facilitating a comprehensive genotype-to-phenotype workflow

Sequence logos are multiple sequence alignment graphs of DNA bases or amino acids [28]. In protein sequence logo figures, protein sequence amino acids are represented with a one letter code stacked at each position to indicate the frequency (by height) of each amino acid found in that position; the total height of the stack reflects the degree of sequence conservation for that position. To further aid KBCommons web portal users in their genomic inquiries, the Protein Sequence Logos tool was added to the SorghumHub (Fig. 5; Supplemental Figure S1) for quickly analyzing amino acid sequence conservation for protein missense changes identified in the Sorghum Allele Catalog Tool or elsewhere. The sorghum protein sequence logos were precomputed into multiple sequence alignments after blastp for the top 100 National Center for Biotechnology Information (NCBI) nonredundant database hits against each annotated gene in the reference, in this case *Sorghum bicolor* v5.1 using the Amino Conserve Atlas pipeline (https://github.com/AnserM/PSL/). Each protein sequence is aligned with amino acid numbering relative to the reference gene start site, anchored to the *Sorghum bicolor* v5.1 reference positions; any gaps introduced for alignment do not disrupt the reference sequence coordinates. This was done to put the protein sequence logos scale in line with the individual crop reference protein amino acid sequence and the Allele Catalog results, rather than relying on a consensus coordinate scheme. Therefore, each protein starts with a sorghum methionine at amino acid position one, and any gaps do not count against the coordinate positions. Tool users can access protein sequence logos for any annotated sorghum gene ID, view the sequence logo image, protein amino acid sequence, or multiple sequence alignments in FASTA format, all with sorghum as the reference. Additionally, there is an option to download one or more sequence logos by entering the gene names. Clickable links below open a new tab to view genes on SorghumBase or the Sorghum Allele Catalog Tool.

### Case study 1: novel allelic variation in the predicted sorghum flowering time gene SbFT12

Flowering time is a critical agronomic trait that determines a sorghum plant’s adaptation to its environment. For bioenergy sorghums, a key breeding goal is delayed flowering to extend the vegetative phase and maximize biomass yield. While several quantitative trait loci (QTLs) and genes are known to influence flowering time in sorghum, the molecular mechanisms are not fully characterized [29–34]. The Flowering Locus T (FT)-like gene family, part of the phosphatidylethanolamine-binding protein (PEBP) family, are central integrators of environmental and developmental signals that promote flowering. In sorghum, the thirteen FT-like genes (*SbFT1*-*13*) include promising candidates for natural variation in flowering time [35]. Among these, *SbFT12* (Sobic.006G047700) is predicted to encode a 185-amino acid protein with significant sequence similarity to known floral activators: 57% to maize *ZCN8* (*Zea mays* CENTRORADIALIS 8) and 59% to rice *Hd3a* (Heading date 3a). Previous studies have reported non-coding variants in *SbFT12* but none predicted to alter the protein product [36].

**Fig. 6:**
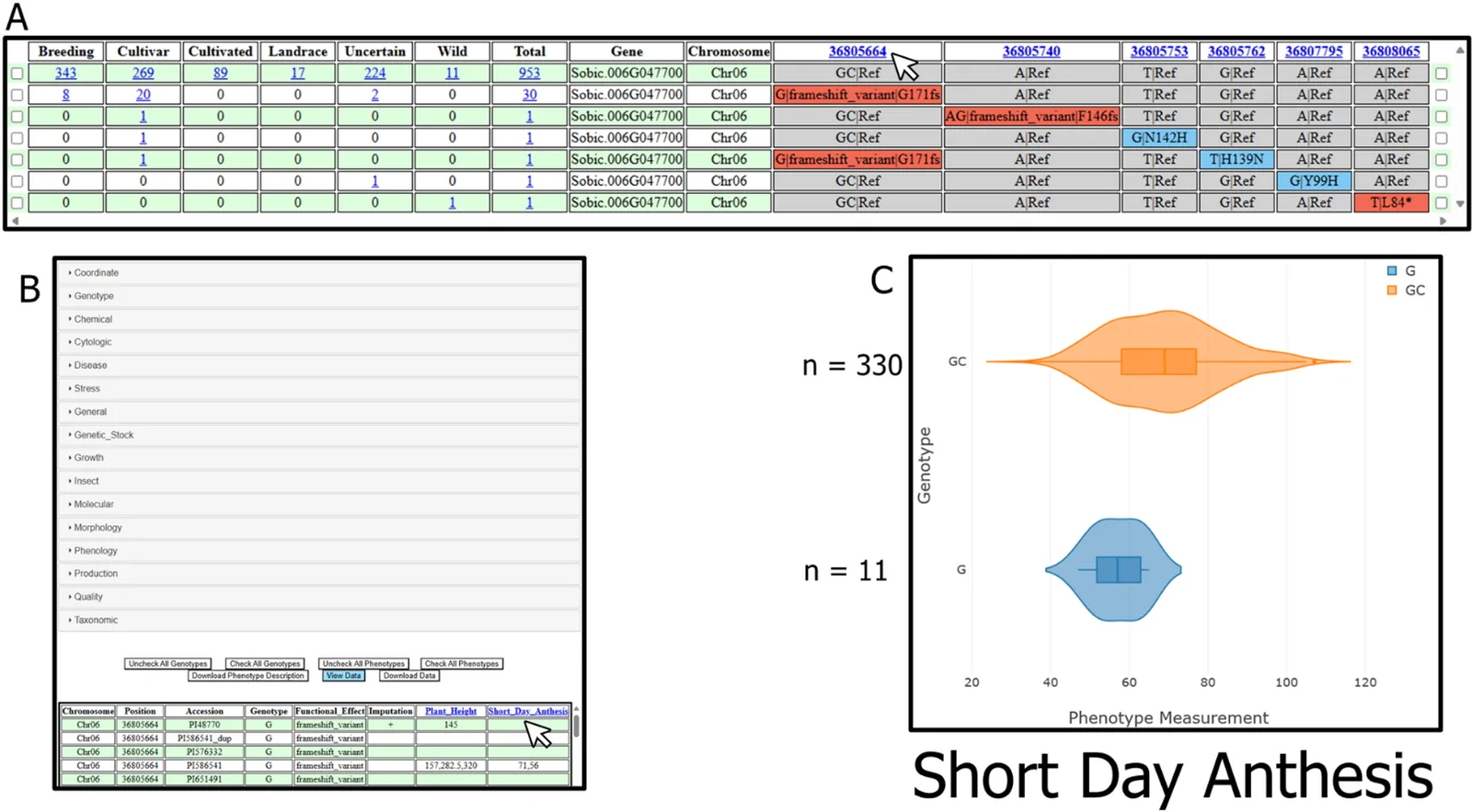
Genotype-phenotype association analysis for Short Day Anthesis for the flowering time gene SbFT12 (Sobic.006G047700) in the Sorghum Allele Catalog Tool. (**A**) The allele catalog data selection interface allows users to filter germplasm based on specific modifying variant positions (allele-defining positions of SbFT12). (**B**) Quantitative and qualitative traits of interest (e.g. "Plant Height" and “Short Day Anthesis”) were selected to be viewed in an interactive table or downloaded for further analysis. (**C**) Violin plots generated by the tool visualize the distribution of a key agronomic trait across the selected alleles of SbFT12. "Short Day Anthesis" (a measure of flowering time) shows a clear separation between alleles, with deletion of cytosine at position Chr06:36805664 resulting in frameshift variant G171fs that could be associated with earlier flowering (approx. 57 days) than those with the reference allele (approx. 69 days). A t-test confirmed significance at p-value<0.005

Interrogation of the Sorghum Allele Catalog for *SbFT12* (Sobic.006G047700) in the Sbicolor988v5.1 dataset revealed seven distinct alleles, illustrating substantial genetic diversity at this locus (Fig. 6A). The reference allele (from Tx623) was predominant, found in 953 accessions. Strikingly, we identified 35 accessions carrying alleles with predicted high-impact variants. The second most frequent allele was defined by a single base deletion of a cytosine at Chr06:36,805,664, predicted to cause a frameshift mutation at glycine-171 (G|frameshift_variant|G171fs). This allele is found in 31 accessions, the majority of which (20 cultivars, 8 breeding lines) are improved germplasm, suggesting its potential agronomic relevance. Other rare, high impact alleles discovered include a guanine insertion at Chr06:36,805,740 that is predicted to cause a frameshift at phenylalanine-146 (AG|frameshift_variant|F146fs) in the cultivar PI329480. A wild accession, PI535995, was found to carry a nonsense mutation (A to T) at Chr06:36,808,065, predicted to introduce a premature stop codon at leucine-84 (L84*). Three independent missense variants were also identified: Y99H (G|Y99H) in the wild accession PI369487, H139N (T|H139N) in cultivar PI653616, and N142H (G|N142H) in cultivar PI452692. The accession PI653616 was unique in carrying both the H139N missense and the G171fs frameshift variants. Given the high frequency of the G171fs allele, we investigated its phenotypic effect. Since *SbFT12* is a predicted floral activator, we analyzed its association with the phenology trait “Short Day Anthesis.” From the 31 accessions with the G171fs allele, 11 had curated phenotypic data available from GRIN (Fig. 6B). Comparison with 330 accessions carrying the reference allele revealed a phenotypic difference: accessions with the SbFT12 predicted loss-of-function G171fs allele flowered significantly earlier (approximately 12 days, *t*-test calculation *p-*value < 0.005) than those with the reference allele (Fig. 6C). The agronomic trait “Plant Height” also showed an association with a similar analysis for the *SbFT12* G171fs allele in which accessions with the frameshift genotype were on average 37 cm shorter than those with the reference allele, (*t*-test calculation *p-*value < 0.014) (Supplemental Figure S2). This suggests *SbFT12* may act as a floral repressor in certain genetic backgrounds, or that the truncated protein retains or alters function.

**Fig. 7:**
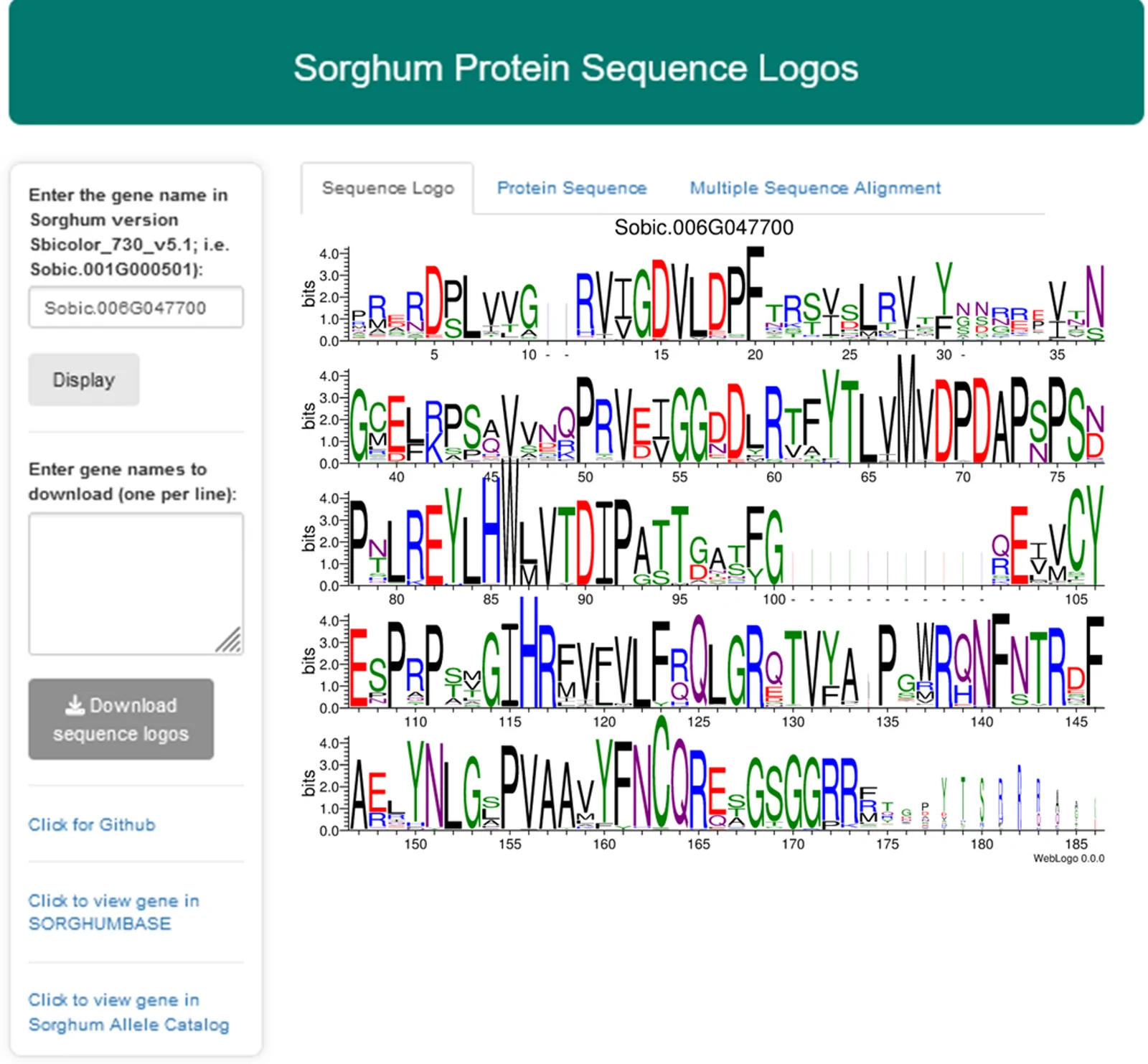
Case study: Visualizing allele-driven protein variation in the flowering time regulator *SbFT12. *This visualization reveals positions critical for phosphatidylethanolamine-binding protein (PEBP) family function such as active sites or ligand-binding residues with tall, invariant stacks. In contrast, variable regions (shorter, multi-letter stacks) may represent neutral variation or functionally important divergence between species. The leucine at position 84 (L84) is highly conserved, indicating that a nonsense mutation (L84*) at this site is likely deleterious. The logo provides evolutionary context for assessing the impact of other discovered missense (Y99H, H139N, N142H) and frameshift (F146fs, G171fs) variants

To evolutionarily contextualize these variants, we generated a protein sequence logo for Sobic.006G047700 using the Sorghum Protein Sequence Logos tool (Fig. 7). For this protein sequence logo, the start methionine at amino acid position one was not conserved, or the Tx623 version 5.1 annotation does not identify the authentic start codon for this gene. The analysis revealed that the leucine at position 84 resides in a highly conserved region, indicating that the predicted L84* nonsense mutation could result in an amorphic and dysfunctional protein severely truncated, missing over half of the polypeptide. The logo also provided context for the missense variants. Tyrosine-99 (Y99) is not perfectly conserved (phenylalanine is the consensus), a change to histidine (Y99H) represents a shift from a hydrophobic to a positively charged residue. The histidine at position 139 (H139) is conserved, and its change to the polar, uncharged asparagine (H139N) could impact local charge and structure. Similarly, the N142H mutation substitutes a polar, uncharged asparagine for a positively charged histidine in a position that typically conserves polar, uncharged residues (asparagine or serine). The frameshifts at phenylalaine-146 and glycine-171 are nearer to the C-terminal end of the predicted 185 amino acid SbFT12 polypeptide. The glycine-171 frameshift deletion occurs only 14 amino acids from the C-terminal end. The predicted frameshift at phenylalaine-146 would impact almost a quarter of all amino acids in the protein. Together, these analyses highlight the utility of the Sorghum Allele Catalog and Protein Sequence Logos tools for rapidly identifying and contextualizing genetic variation across diverse sorghum accessions. By linking predicted high-impact alleles in candidate genes like *SbFT12* to curated phenotypic data, researchers can efficiently prioritize variants for functional validation and breeding, accelerating the discovery of key regulators in complex traits such as flowering time.

### Case study 2: allele mining the sorghum betaine aldehyde dehydrogenase (BADH2) gene responsible for fragrance

**Fig. 8:**
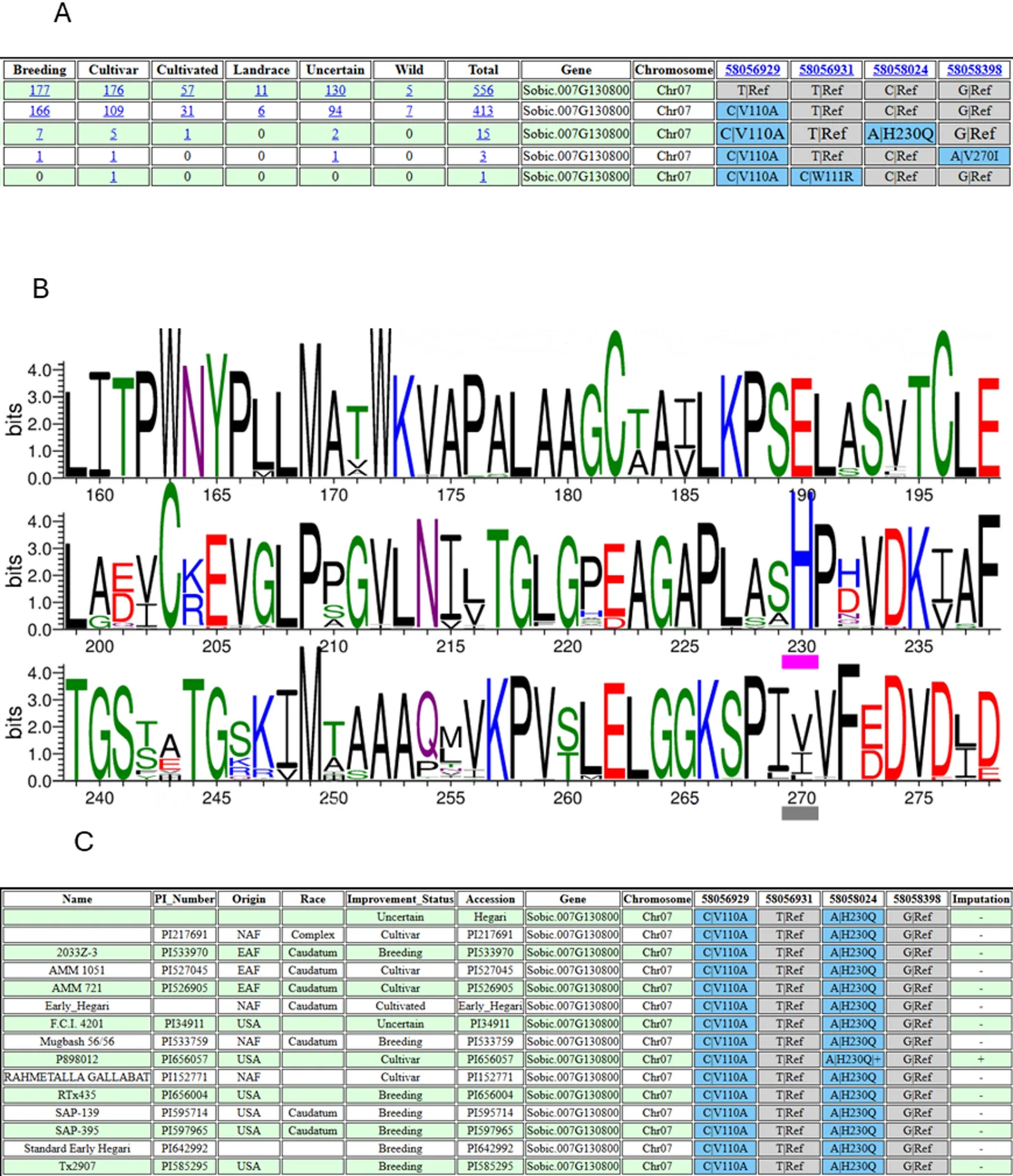
SorghumHub tool analyses of the betaine aldehyde dehydrogenase (BADH2) gene responsible for fragrance. (**A**) Sorghum Allele Catalog results for Sobic.007G130800. (**B**) Sorghum Protein Sequence Logos result for amino acid conservation between position 159 and 278 for Sobic.007G130800. Amino acid position 230 is indicated by pink underline; position 270 is indicated by gray underline. (**C**) Metadata for the fifteen accessions carrying the H230Q allele, showing three accessions with ‘Hegari’ in their name. The imputation status for each variant is indicated by a “+” symbol

Palatability in foods and feeds is influenced in part by volatile compounds such as 2-acetyl-1-pyrroline (2AP) [9]. The potent fragrant aromatic 2AP accumulates when betaine aldehyde dehydrogenase (BADH2) enzymes are dysfunctional in rice and other crop species including sorghum [9, 10, 37]. The sorghum *BADH2* paralog is Sobic.007G130800 in the sense orientation in the genome; a fragrant sorghum accession was characterized to have an almost 1.5 kilobasepair (kb) deletion that eliminated several exons of the gene [10]. Sorghum Allele Catalog analysis of Sobic.007G130800 in the Sbicolor988v5.1 dataset did not reveal the 1.5 kb allele, but there were four non-reference alleles present in the dataset that were defined by one or two missense variants (Fig. 8A). The reference allele was most frequent (56%) and spread across all improvement status subcategories. The allele defined by only a valine to alanine (both nonpolar/hydrophobic) missense change at amino acid position 110 (V110A) was found in about 42% of accessions; the V110A change was not conserved as alanine was the most frequent amino acid at position 110 (Supplemental Figure S3). Fifteen sorghum accessions had an allele with both the V110A change and a missense change of histidine to glutamine at position 230 (H230Q). Histidine is polar and positively charged, while glutamine is polar and uncharged. The Sorghum Protein Sequence Logo result for Sobic.007G130800 revealed the histidine at amino acid 230 was a highly evolutionarily conserved position surrounded by a region of mixed amino acid conservation (Fig. 8B). The metadata popout details of the fifteen accessions with the H230Q-defining *BADH2* allele shows three accessions with ‘Hegari’ in their name (Fig. 8C). The imputation status for each variant position is demarked with a “+” in the imputation column as well as a “+” in the variant cell, as was described for the original Allele Catalog Tool [14]. There were three accessions with both the V110A change and a missense change of valine to isoleucine at amino acid 270 (V270I), but valine and isoleucine are nonpolar hydrophobic amino acids that were both present at position 270 in the Sorghum Protein Sequence Logo result (Fig. 8A and B). The accession PI 302252 had both the V110A change and a missense change of tryptophan (nonpolar aromatic) to arginine (polar and positively charged) at amino acid 111 (W111R); the Sorghum Protein Sequence Logo result indicated amino acid position 111 was a highly evolutionarily conserved tryptophan in a region of mixed amino acid conservation (Supplemental Figure S3).

## Discussion

For many taxa, next generation sequencing information exists for at least one reference genome and up to several thousand samples. However, this vast resource of WGRS data is frequently inaccessible at the gene level for further use due to a lack of tools to manage, access, and connect the data to accessions or traits. Several major crop species have established genomic “hubs”; however, these sites are often static and species-specific. These genomic “hubs” or “bases” can be found for major crops like soybean (SoyBase) or rice (RiceBase) whose platforms tend to be static in nature, focusing on a core reference genome or curated markers [38, 39]. SorghumBase (sorghumbase.org) has evolved to give new life to otherwise orphaned datasets in the form of a pan-genomics browser [40] with genetic variant information and various dataset connectivity linking to other databases. SorghumBase is the current gold standard genomic “base” for sorghum. However, despite offering valuable links to Genbank records, SorghumBase lacks dynamic tools to directly connect gene-based variants with phenotypic traits from GRIN accessions. Here we demonstrated the new SorghumHub provides an exploration platform for mining alleles for sorghum genes of interest. Our tools focus on gene regions and enable connecting variants to sorghum phenotype observations recorded from several curated GRIN studies. The SorghumHub is a part of the KBCommons ecosystem that is meant to complement rather than replace SorghumBase, as is the case for the SoyHub and SoyBase.

The Sorghum Allele Catalog Tool and related tools, such as the Protein Sequence Logos, offer a complementary and novel solution by enabling researchers to explore gene-based variation organized into actual alleles rather than focusing on individual SNPs or InDels. This gene-centric approach emphasizes functionally meaningful combinations of variants that define an allele, streamlining analyses that link genetic diversity to observable traits. For instance, researchers can directly search gene models like *SbFT12* and compare functional alleles across accessions with documented flowering times, bypassing the need for complex variant parsing or whole genome association studies. This significantly lowers the barrier to entry for applied genomics, particularly for under-resourced research groups or interdisciplinary scientists unfamiliar with bioinformatics pipelines.

Unlike SorghumBase, which currently does not prioritize fine-grained variant-to-phenotype exploration, SorghumHub was designed to be exploratory and flexible. It enables users to mine alleles of interest quickly, assess their predicted impact, and connect them to curated phenotypic data from GRIN and published studies. By integrating functional annotations and providing intuitive visualizations such as protein sequence logos, SorghumHub helps users distinguish potentially causal variants from background noise. These logos illustrate evolutionary conservation at each amino acid position with a coordinate system anchored to the sorghum reference, offering key insights into the biological relevance of observed mutations, a major advantage over standard SNP catalogs that do not consider protein structure or function.

The abundance of variant positions in any re-sequenced population can overwhelm researchers trying to identify functionally important mutations. Our gene and allele-based Allele Catalog tool addresses this by highlighting only the modifying effect variants, which are the most biologically relevant to most breeders and geneticists. The Sorghum Allele Catalog uniquely supports this approach by tracking full alleles with defined protein modifying consequences, or the combinations of changes across a gene, rather than isolating single genetic variants. This supports use cases such as confirming a candidate allele’s association with phenotypic traits, narrowing targets for functional validation, or identifying novel genetic sources of adaptation.

In real world research applications, this platform aids not only in allele mining and hypothesis generation following GWAS but also provides valuable information to guide in validating candidate genes identified through evolutionary genomics or expression studies [41]. The ability to connect functional variation to accession level phenotype data accelerates applied breeding decisions, especially when phenotypes are already recorded in germplasm collections. For example, the identification of the predicted G171fs variant in *SbFT12* and its association with early flowering demonstrates the value of integrating predicted protein disruption with observed trait data, all within a few exploratory clicks. Similarly, the identification of novel alleles of the aroma gene *BADH2* provides accessions to characterize further for loss of gene function and previously unknown sources of potentially fragrant sorghum, a desired trait [9].

Ultimately, sites like SorghumHub or SoyHub highlight the growing need for dynamic, gene-based genotype to phenotype platforms tailored for each species. As more species-specific genebanks adopt genomic characterization, such platforms will be essential to unlocking the full potential of existing sequence data, especially in translating legacy datasets into actionable knowledge for breeding, adaptation, and crop improvement research.

## Conclusions

Advancements in high-throughput sequencing and bioinformatics have revolutionized crop genomics, yet species-specific challenges persist in translating genomic data into applicable insights for crop improvement. Many crops lack tailored and enduring genomic resources, such as high-quality reference genomes, annotated gene networks, or genotype-phenotype association databases, limiting precision breeding and functional genomics applications. This research addresses these gaps by developing a hub optimized for individual crop species, integrating computational pipelines to develop user friendly tools. We demonstrate this in *Sorghum bicolor*, where candidate genes and their alleles can be explored effortlessly and compared to phenotypes or other species for comparative studies. Missense changes can be prioritized with efficient analyses of amino acid sequence conservation. By bridging the gap between genomics and breeding, this approach can help accelerate the development of abiotic and biotic resilient, value-added high-yielding crops.

## Supplementary Information

Supplementary Material 1.

## Data Availability

The datasets supporting the conclusions of this article are included within the article (and its additional files), at the SoyKB web portal/KBCommons web portal, (https://kbcommons.org/system/home/Sbicolor/SorghumHub) and Github (links cited within text).

## Abbreviations

CNV: Copy Number Variant
ENA: European Nucleotide Archive
FT: Flowering Time
FTL: Flowering Time-Like
FTP: File Transfer Protocol
GFF: General Feature Format
GRIN: Germplasm Resource Information Network
GWAS: Genome Wide Association Study
MSA: Multiple Sequence Alignment
NASS: National Agricultural Statistics Service
NCBI: National Center for Biotechnology Information
NPGS: National Plant Germplasm System
QTL: Quantitative Trait Locus
TERRA-REF: Transportation Energy Resources from Renewable Agriculture Phenotyping Reference
USDA: United States Department of Agriculture
VCF: Variant Call Format
WGRS: Whole Genome Resequencing
