# Supplementary material for: SorghumHub: featuring the next phase for the KBCommons web portal with new molecular biology features
Source: BMC Genomics. 2026 Jul 16;27:840. doi: 10.1186/s12864-026-13113-y (PMC13656370; doi:10.1186/s12864-026-13113-y)
Supplement: Supplementary file 1 [file 12864_2026_13113_MOESM1_ESM.docx]

Supplemental Tables and Figures

Supplemental Table 1. Sorghum988 metadata.

| Accession | Name | PI_Number | Origin | Race | Improvement_Status |
| --- | --- | --- | --- | --- | --- |
| 80M |  |  |  | Durra | Breeding |
| Ai4 | Ai4 |  | EAS | Complex | Cultivated |
| AusTRCF_317961 | AusTRCF_317961 |  | AUS | Wild | Wild |
| B35 | B35 |  | EAF | Durra | Uncertain |
| B923296 | B923296 |  | AUS | Complex | Cultivated |
| B963676 | B963676 |  | AUS | Complex | Cultivated |
| BTx623 | BTx623 |  | USA |  | Breeding |
| BTx642 |  |  |  |  | Breeding |
| Cherekit_IBC_E-460 |  |  | EAF | Caudatum | Landrace |
| Early_Hegari | Early_Hegari |  | NAF | Caudatum | Cultivated |
| E-Tian |  |  | EAS | Kafir | Uncertain |
| GRIF16309 | GRIF16309 |  | EAF | Wild | Wild |
| Hegari |  |  |  |  | Uncertain |
| IBC_E-38432 |  |  | EAF | Durra | Landrace |
| ICSV745 | ICSV745 |  | SAS | Caudatum | Cultivated |
| IS3614-2 | IS3614 |  | WAF | Guinea | Landrace |
| IS3620C |  |  |  | Guinea | Uncertain |
| IS8525 | BUTHA |  | EAF | Kafir | Landrace |
| IS9710 | EL LUBEI |  | NAF | Complex | Landrace |
| Ji2731 |  |  | EAS | Complex | Uncertain |
| Karper_669 | Karper_669 |  | USA | Complex | Cultivated |
| Keller |  |  | USA | Kafir | Uncertain |
| Kilo_IBC_E-382 |  |  | EAF | Durra | Wild |
| KS115 | KS115 |  | USA | Complex | Cultivated |
| LR9198 | LR9198 |  | EAS | Complex | Cultivated |
| M35-1 | M35-1 |  | SAS | Complex | Cultivar |
| Macia | MACIA |  | EAF | Caudatum | Cultivar |
| NSL102183 | NSL102183 |  | LAC | Caudatum | Uncertain |
| NSL365694 | Line 407 |  | WAF | Kafir | Uncertain |
| NSL365712 | Safra |  | NAF | Durra | Uncertain |
| NSL50448 | IS1159 |  | WAF | Guinea | Uncertain |
| NSL50449 | IS1166 |  | EAF | Guinea | Uncertain |
| NSL50464 | IS3829 |  | WAF | Guinea | Uncertain |
| NSL50499 | IS6729 |  | WAF | Guinea | Uncertain |
| NSL50613 | IS7541 |  | WAF | Guinea | Uncertain |
| NSL50614 | NSL50614 |  | WAF | Guinea | Uncertain |
| NSL50649 | NSL50649 |  | WAF | Guinea | Uncertain |
| NSL50663 | IS7617 |  | WAF | Guinea | Uncertain |
| NSL50685 | IS7668 |  | WAF | Guinea | Uncertain |
| NSL50716 | IS7714 |  | WAF | Guinea | Uncertain |
| NSL50748 | IS7786 |  | WAF | Guinea | Uncertain |
| NSL50843 | IS7947 |  | WAF | Guinea | Uncertain |
| NSL51356 | IS5457 |  | SAS | Kafir | Uncertain |
| NSL51902 | IS3623 |  | WAF | Caudatum | Uncertain |
| NSL51963 | IS7449 |  | WAF | Caudatum | Uncertain |
| NSL51969 | IS7528 |  | WAF | Caudatum | Uncertain |
| NSL51981 | IS7787 |  | WAF | Guinea | Uncertain |
| NSL52282 | IS5821 |  | SAS | Kafir | Uncertain |
| NSL54114 | IS7762 |  | WAF | Guinea | Uncertain |
| NSL54141 | NSL54141 |  | WAF | Guinea | Uncertain |
| NSL54162 | IS7720 |  | WAF | Guinea | Uncertain |
| NSL54165 | IS7736 |  | WAF | Guinea | Uncertain |
| NSL54175 | IS7198 |  | WAF | Guinea | Uncertain |
| NSL54184 | IS969 |  | EAF | Guinea | Uncertain |
| NSL54219 | IS7502 |  | WAF |  | Uncertain |
| NSL54221 | IS7518 |  | WAF | Guinea | Uncertain |
| NSL54222 | IS7524 |  | WAF | Guinea | Uncertain |
| NSL54231 | IS7436 |  | WAF |  | Uncertain |
| NSL54236 | IS7542 |  | WAF | Guinea | Uncertain |
| NSL54297 | Balangiva mwanga |  | EAF | Caudatum | Uncertain |
| NSL54318 | IS7367 |  | WAF | Guinea | Uncertain |
| NSL54577 | IS7223 |  | WAF | Durra | Uncertain |
| NSL54591 | IS1456 |  | SAS | Durra | Uncertain |
| NSL54705 | IS2206 |  |  |  | Uncertain |
| NSL54779 | IS4540 |  | SAS | Durra | Uncertain |
| NSL55032 | IS5677 |  | SAS | Durra | Uncertain |
| NSL55671 | IS5839 |  | SAS | Durra | Uncertain |
| NSL55725 | IS6398 |  | SAS | Durra | Uncertain |
| NSL55731 | IS6405 |  | SAS | Durra | Uncertain |
| NSL55749 | IS8134 |  | EAF | Caudatum | Uncertain |
| NSL67916 | IS6426 |  | SAS | Durra | Uncertain |
| NSL67921 | NSL67921 |  | SAS | Durra | Uncertain |
| PI144134 | Inyangentombi | PI144134 | SAF |  | Cultivar |
| PI145619 | ISIDOMBA | PI145619 | SAF | Caudatum | Cultivar |
| PI145626 | Manyoble | PI145626 | SAF | Kafir | Cultivar |
| PI145632 | TEGEVINI | PI145632 | SAF |  | Cultivar |
| PI145633 | Tugela Ferry | PI145633 | SAF |  | Cultivar |
| PI146890 | SUGAR DRIP | PI146890 | SAF |  | Cultivar |
| PI147224 |  | PI147224 | SAS |  | Cultivar |
| PI147828 | Jehlde No. 22 | PI147828 | EAF | Durra | Uncertain |
| PI147838 | SUMAC ACC 2 | PI147838 | EAF | Durra | Uncertain |
| PI147933 | Maho Verro No. 30 | PI147933 | EAF | Durra | Uncertain |
| PI147937 | Tinkush | PI147937 | EAF | Durra | Uncertain |
| PI147938 | Uki | PI147938 | EAF | Durra | Uncertain |
| PI147939 | Zangada Tinkush | PI147939 | EAF | Durra | Uncertain |
| PI148083 | Adesh | PI148083 | EAF | Durra | Uncertain |
| PI148084 | Amelsie | PI148084 | EAF | Durra | Uncertain |
| PI148084_dup | Amelsie_dup | PI148084_dup | EAF | Durra | Uncertain |
| PI148084_dup2 | Amelsie_dup2 | PI148084_dup2 | EAF | Durra | Uncertain |
| PI148086 | No. 73 | PI148086 | EAF | Durra | Uncertain |
| PI148089 | Buketie | PI148089 | EAF | Durra | Uncertain |
| PI148089_dup | Buketie_dup | PI148089_dup | EAF | Durra | Uncertain |
| PI148089_dup2 | Buketie_dup2 | PI148089_dup2 | EAF | Durra | Uncertain |
| PI148101 | Kebedjie | PI148101 | EAF | Durra | Uncertain |
| PI148114 | IS 12561 | PI148114 | EAF | Durra | Uncertain |
| PI149831 | IS 2463 | PI149831 | EAF | Caudatum | Uncertain |
| PI151198 | IS 12562 | PI151198 | EAF | Durra | Uncertain |
| PI152591 |  | PI152591 | EAF | Caudatum | Cultivar |
| PI152594 | ANKOLIB KHAFIF | PI152594 | NAF | Durra | Uncertain |
| PI152636 | FETERITA FAYOUMI D.S. 18 | PI152636 | NAF | Caudatum | Uncertain |
| PI152651 | Feterita Geshaish | PI152651 | NAF |  | Breeding |
| PI152654 | FETERITA HARIG 2 | PI152654 | NAF |  | Uncertain |
| PI152694 | Kafir Slongi | PI152694 | NAF |  | Cultivar |
| PI152702 | KLOR | PI152702 | NAF | Caudatum | Uncertain |
| PI152705 | Kokla | PI152705 | NAF | Caudatum | Uncertain |
| PI152727 |  | PI152727 | EAF |  | Cultivar |
| PI152728 | MANALEK | PI152728 | NAF | Durra | Cultivar |
| PI152728_dup |  | PI152728_dup | NAF | Durra | Cultivar |
| PI152730 | MATCHIKAH 1 | PI152730 | NAF | Caudatum | Uncertain |
| PI152733 |  | PI152733 | NAF | Caudatum | Cultivar |
| PI152748 | NYAN DOK | PI152748 | NAF | Caudatum | Uncertain |
| PI152751 | NYTWAL | PI152751 | NAF | Guinea | Cultivar |
| PI152751_dup |  | PI152751_dup | NAF | Guinea | Cultivar |
| PI152771 | RAHMETALLA GALLABAT | PI152771 | NAF |  | Cultivar |
| PI152816 | WAD FUR WHITE | PI152816 | NAF |  | Cultivar |
| PI152816_dup |  | PI152816_dup | NAF |  | Cultivar |
| PI152828 | U.T. 23 | PI152828 | SAF |  | Cultivar |
| PI152860 | MERASI | PI152860 | NAF |  | Cultivar |
| PI152862 | KULUM BROWN | PI152862 | NAF |  | Cultivar |
| PI152923 | Duro El Jack | PI152923 | NAF |  | Cultivar |
| PI152961 | MALNAL | PI152961 | NAF | Caudatum | Cultivar |
| PI152963 | Thok (B) | PI152963 | NAF |  | Cultivar |
| PI152965 | TADA | PI152965 | NAF |  | Cultivar |
| PI152966 | Ayuak | PI152966 | NAF |  | Cultivar |
| PI152967 | WERAKAN | PI152967 | NAF | Guinea | Cultivar |
| PI152971 | AWANLEK | PI152971 | NAF |  | Cultivar |
| PI152971_dup |  | PI152971_dup | NAF |  | Cultivar |
| PI153824 | MORITI | PI153824 | EAF | Caudatum | Uncertain |
| PI153844 | MUHIA | PI153844 | EAF | Caudatum | Uncertain |
| PI153852 | MZEKWA 2 | PI153852 | EAF | Caudatum | Uncertain |
| PI153860 | NGARARE, NZEKWA | PI153860 | EAF | Caudatum | Uncertain |
| PI153877 |  | PI153877 | EAF |  | Cultivar |
| PI154846 |  | PI154846 | EAF |  | Cultivar |
| PI154944 | L31 Emiroit | PI154944 | EAF |  | Cultivar |
| PI154987_redo |  | PI154987_redo | SAF |  | Uncertain |
| PI154988 |  | PI154988 | SAF |  | Cultivar |
| PI155149 | Dhurra No. 7 | PI155149 | SEEU_ME |  | Cultivar |
| PI155516 | MASAKA | PI155516 | EAF | Guinea | Cultivar |
| PI155760 | Namuse | PI155760 | EAF |  | Cultivar |
| PI155885 | IS 12598 | PI155885 | EAF | Bicolor | Cultivar |
| PI156018 | MN 1615 | PI156018 | SAF | Guinea | Cultivar |
| PI156178 |  | PI156178 | EAF | Caudatum | Cultivar |
| PI156203 |  | PI156203 | EAF | Caudatum | Cultivar |
| PI156217 | MN 2109 | PI156217 | EAF | Durra | Cultivar |
| PI156326 | CHIMPAMBA | PI156326 | EAF | Caudatum | Cultivar |
| PI156330 |  | PI156330 | EAF | Durra | Cultivar |
| PI156393 |  | PI156393 | EAF | Caudatum | Cultivar |
| PI156463 | Dobbs | PI156463 | EAF | Caudatum | Cultivar |
| PI156487 |  | PI156487 | EAF | Guinea | Cultivar |
| PI156871 | Rutobo | PI156871 | SAF | Caudatum | Cultivar |
| PI156890 | Dura Huria | PI156890 | SAF |  | Cultivar |
| PI157030 | Andiwo III 57 | PI157030 | EAF | Caudatum | Cultivar |
| PI157033 | Ifube No. 18 | PI157033 | EAF | Caudatum | Cultivar |
| PI157035 | Nyagwang No. 56 | PI157035 | EAF |  | Cultivar |
| PI157804 | Feterita Abu Derega | PI157804 | NAF |  | Cultivar |
| PI167093 | IS 81 | PI167093 | NAF | Durra | Cultivar |
| PI170787 | MN 2826 | PI170787 | SEEU_ME | Caudatum | Cultivar |
| PI17548 | RED AMBER | PI17548 | AUS |  | Breeding |
| PI175919 |  | PI175919 | SEEU_ME | Caudatum | Cultivar |
| PI176766 |  | PI176766 | SEEU_ME | Bicolor | Cultivar |
| PI179749 |  | PI179749 | SAS | Durra | Cultivar |
| PI180348 | Juar | PI180348 | SAS | Durra | Cultivar |
| PI180348_dup |  | PI180348_dup | SAS | Durra | Cultivar |
| PI181080 | HONEY SORGHUM | PI181080 | SAS |  | Cultivar |
| PI181083 | KAMANDRI | PI181083 | SAS | Durra | Cultivar |
| PI181899 | IS 12893 | PI181899 | SEEU_ME |  | Cultivar |
| PI185458 | MN 2943 | PI185458 | SAS |  | Uncertain |
| PI185573 |  | PI185573 | SAF | Kafir | Cultivar |
| PI196049 | IS 2131 | PI196049 | EAF | Durra | Cultivar |
| PI196583 | MN 3080 | PI196583 | EAS |  | Cultivar |
| PI196586 | MN 3083 | PI196586 | SAS | Guinea | Cultivar |
| PI196598 | MN 3095 | PI196598 | EAS |  | Cultivar |
| PI197542 |  | PI197542 | NAF | Bicolor | Cultivar |
| PI19770 |  | PI19770 | SAF | Caudatum | Cultivar |
| PI213901 |  | PI213901 | EAF | Wild | Cultivar |
| PI217691 |  | PI217691 | NAF | Complex | Cultivar |
| PI218112 | IS 2352 | PI218112 | SAS | Durra | Cultivar |
| PI221548 | AJEBICHINKA | PI221548 | WAF | Kafir | Cultivar |
| PI221624 | MORI WHITE GLUME | PI221624 | WAF | Guinea | Uncertain |
| PI221651 | UWARETE | PI221651 | WAF | Guinea | Cultivar |
| PI221662 | BONKUM | PI221662 | WAF | Durra | Uncertain |
| PI239439_redo |  | PI239439_redo | SAF | Kafir | Uncertain |
| PI248317 | KARAD-2-7-11 | PI248317 | SAS | Durra | Uncertain |
| PI24969 | DWARF YELLOW MILO | PI24969 | EAS |  | Breeding |
| PI250583 | MN 4125 | PI250583 | NAF | Durra | Cultivar |
| PI251672 | MN 4135 | PI251672 | SEEU_ME |  | Cultivar |
| PI253986 | IS 13150 | PI253986 | SEEU_ME |  | Cultivar |
| PI255744 | MN 4466 | PI255744 | SEEU_ME |  | Cultivar |
| PI257599 | NO. 5 GAMBELA | PI257599 | EAF | Caudatum | Cultivar |
| PI257600 | NO. 6 GAMBELA | PI257600 | EAF | Caudatum | Cultivar |
| PI257600_dup |  | PI257600_dup | EAF | Caudatum | Cultivar |
| PI266927 | CHOLAM | PI266927 | SAS |  | Cultivar |
| PI267542 | IS 2904 | PI267542 | WAF | Guinea | Uncertain |
| PI267573 | IS 2983 | PI267573 | EAF | Durra | Cultivar |
| PI267614 | Chabeady | PI267614 | EAF | Guinea | Uncertain |
| PI267643 | Cullubi | PI267643 | EAF | Durra | Uncertain |
| PI267650 | IS 3063 | PI267650 | EAF | Durra | Uncertain |
| PI267653 | IS 2757 | PI267653 | EAF | Durra | Uncertain |
| PI273969 | MN 4578 | PI273969 | EAF |  | Cultivar |
| PI276770 | IS 13299 | PI276770 | EAF | Durra | Uncertain |
| PI276785 | IS 12625 | PI276785 | EAF | Durra | Uncertain |
| PI276787 | IS 12627 | PI276787 | EAF | Durra | Uncertain |
| PI276789 | IS 12629 | PI276789 | EAF | Durra | Uncertain |
| PI276790 | IS 12630 | PI276790 | EAF | Durra | Uncertain |
| PI276800 | IS 12636 | PI276800 | EAF | Durra | Uncertain |
| PI276801 | IS 12637 | PI276801 | EAF | Caudatum | Uncertain |
| PI276801_dup | IS 12637_dup | PI276801_dup | EAF | Caudatum | Uncertain |
| PI276803 | IS 12638 | PI276803 | EAF | Durra | Uncertain |
| PI276806 | IS 12641 | PI276806 | EAF | Durra | Uncertain |
| PI276816 | IS 12646 | PI276816 | EAF | Durra | Uncertain |
| PI276820 | IS 12650 | PI276820 | EAF | Durra | Uncertain |
| PI276823 | IS 12652 | PI276823 | EAF | Durra | Uncertain |
| PI276830 | IS 12658 | PI276830 | EAF | Durra | Uncertain |
| PI276837 | IS 12661 | PI276837 | EAF | Durra | Cultivar |
| PI276853A | Collubi Dwarf | PI276853A | EAF | Durra | Uncertain |
| PI285039 | IS 12676 | PI285039 | WAF | Caudatum | Uncertain |
| PI285042 | IS 12678 | PI285042 | WAF | Caudatum | Uncertain |
| PI285042_dup | IS 12678_dup | PI285042_dup | WAF | Caudatum | Uncertain |
| PI285042_dup2 | IS 12678_dup2 | PI285042_dup2 | WAF | Caudatum | Uncertain |
| PI285043 | IS 12679 | PI285043 | WAF | Guinea | Uncertain |
| PI291231 | N-12 | PI291231 | SAS | Durra | Uncertain |
| PI291239 | IS 1598 | PI291239 | SAS | Durra | Uncertain |
| PI291240 | K-2 Cholam | PI291240 | SAS | Durra | Uncertain |
| PI291246 | IS 2253 | PI291246 | LAC | Durra | Cultivar |
| PI297117 | IS 13600 | PI297117 | EAF | Caudatum | Uncertain |
| PI300118 |  | PI300118 | SAF | Wild | Cultivar |
| PI300119 | 329 | PI300119 | SAF | Wild | Cultivar |
| PI302221 |  | PI302221 | SEEU_ME | Wild | Cultivar |
| PI302252 |  | PI302252 | EAS |  | Cultivar |
| PI303658 | Nerum Boer | PI303658 | NAF |  | Cultivar |
| PI329250 | ADAR | PI329250 | EAF | Wild | Wild |
| PI329252 | IS 11010 | PI329252 | EAF | Wild | Wild |
| PI329256 | IS 11014 | PI329256 | EAF |  | Cultivar |
| PI329286 | IS 11044 | PI329286 | EAF |  | Cultivar |
| PI329299 | IS 11057 | PI329299 | EAF |  | Cultivar |
| PI329300 |  | PI329300 | EAF |  | Cultivar |
| PI329301 |  | PI329301 | EAF |  | Cultivar |
| PI329310 | IS 11068 | PI329310 | EAF |  | Cultivar |
| PI329319 | IS 11077 | PI329319 | EAF |  | Cultivar |
| PI329326 | IS 11083 | PI329326 | EAF |  | Cultivar |
| PI329333 | IS 11090 | PI329333 | EAF |  | Cultivar |
| PI329338 | IS 11096 | PI329338 | EAF |  | Cultivar |
| PI329351 | IS 11108 | PI329351 | EAF |  | Cultivar |
| PI329394 | IS 11151 | PI329394 | EAF |  | Cultivar |
| PI329403 |  | PI329403 | EAF |  | Cultivar |
| PI329435 | Mashila | PI329435 | EAF |  | Breeding |
| PI329440 | Mashila | PI329440 | EAF |  | Cultivar |
| PI329465 | IS 11222 | PI329465 | EAF |  | Cultivar |
| PI329466 |  | PI329466 | EAF |  | Cultivar |
| PI329471 | IS 11228 | PI329471 | EAF |  | Cultivar |
| PI329478 |  | PI329478 | EAF |  | Cultivar |
| PI329480 | IS 11236 | PI329480 | EAF |  | Cultivar |
| PI329501 |  | PI329501 | EAF |  | Cultivar |
| PI329506 | IS 11260 | PI329506 | EAF |  | Cultivar |
| PI329510 | IS 11264 | PI329510 | EAF |  | Cultivar |
| PI329511 | IS 11265 | PI329511 | EAF |  | Cultivar |
| PI329517 | IS 11269 | PI329517 | EAF |  | Cultivar |
| PI329518 | IS 11270 | PI329518 | EAF |  | Cultivar |
| PI329518_redo |  | PI329518_redo | EAF |  | Cultivar |
| PI329519 | IS 11271 | PI329519 | EAF |  | Cultivar |
| PI329545 |  | PI329545 | EAF |  | Cultivar |
| PI329546 |  | PI329546 | EAF |  | Cultivar |
| PI329550 |  | PI329550 | EAF |  | Cultivar |
| PI329551 |  | PI329551 | EAF |  | Cultivar |
| PI329554 |  | PI329554 | EAF |  | Cultivar |
| PI329569 | IS 11320 | PI329569 | EAF |  | Cultivar |
| PI329570 | IS 11321 | PI329570 | EAF |  | Cultivar |
| PI329573 |  | PI329573 | EAF |  | Cultivar |
| PI329583 |  | PI329583 | EAF |  | Cultivar |
| PI329584 | IS 11335 | PI329584 | EAF |  | Cultivar |
| PI329585 | IS 11336 | PI329585 | EAF |  | Cultivar |
| PI329605 | IS 11350 | PI329605 | EAF |  | Cultivar |
| PI329614 |  | PI329614 | EAF |  | Cultivar |
| PI329615 |  | PI329615 | EAF |  | Cultivar |
| PI329617 |  | PI329617 | EAF |  | Cultivar |
| PI329644 |  | PI329644 | EAF |  | Cultivar |
| PI329645 |  | PI329645 | EAF |  | Cultivar |
| PI329646 |  | PI329646 | EAF |  | Cultivar |
| PI329673 |  | PI329673 | EAF |  | Cultivar |
| PI329699 |  | PI329699 | EAF |  | Cultivar |
| PI329702 |  | PI329702 | EAF |  | Cultivar |
| PI329707 | IS 11443 | PI329707 | EAF | Durra | Uncertain |
| PI329710 |  | PI329710 | EAF |  | Cultivar |
| PI329711 |  | PI329711 | EAF |  | Cultivar |
| PI329719 | IS 11455 | PI329719 | EAF | Durra | Uncertain |
| PI329836 | IS 11569 | PI329836 | EAF | Caudatum | Uncertain |
| PI329841 |  | PI329841 | EAF |  | Cultivar |
| PI329843 |  | PI329843 | EAF |  | Cultivar |
| PI329864 |  | PI329864 | EAF |  | Cultivar |
| PI329865 |  | PI329865 | EAF |  | Cultivar |
| PI329915 | Mashila | PI329915 | EAF | Durra | Uncertain |
| PI330070 | IS 11792 | PI330070 | EAF | Durra | Uncertain |
| PI330070_dup | IS 11792_dup | PI330070_dup | EAF | Durra | Uncertain |
| PI330095 | IS 11815 | PI330095 | EAF | Durra | Uncertain |
| PI330098 | IS 11818 | PI330098 | EAF | Durra | Uncertain |
| PI330122 |  | PI330122 | EAF |  | Cultivar |
| PI330167 |  | PI330167 | EAF |  | Cultivar |
| PI330168 |  | PI330168 | EAF |  | Cultivar |
| PI330169 |  | PI330169 | EAF |  | Cultivar |
| PI330181 |  | PI330181 | EAF |  | Cultivar |
| PI330182 |  | PI330182 | EAF |  | Cultivar |
| PI330184 |  | PI330184 | EAF |  | Cultivar |
| PI330185 |  | PI330185 | EAF |  | Cultivar |
| PI330195 |  | PI330195 | EAF |  | Cultivar |
| PI330196 |  | PI330196 | EAF |  | Cultivar |
| PI330199 |  | PI330199 | EAF |  | Cultivar |
| PI330272 | R-244 | PI330272 | EAF | Wild | Wild |
| PI330796 |  | PI330796 | EAF |  | Cultivar |
| PI330803 |  | PI330803 | EAF |  | Cultivar |
| PI330833 |  | PI330833 | EAF |  | Cultivar |
| PI330858 |  | PI330858 | EAF |  | Cultivar |
| PI330877 | IS 12161 | PI330877 | EAF | Durra | Uncertain |
| PI330886 | IS 12170 | PI330886 | EAF | Durra | Uncertain |
| PI330887 | IS 12171 | PI330887 | EAF | Durra | Uncertain |
| PI330897 | IS 12181 | PI330897 | EAF | Durra | Uncertain |
| PI337680 |  | PI337680 | EAF |  | Cultivar |
| PI337689 |  | PI337689 | EAF |  | Cultivar |
| PI34911 | F.C.I. 4201 | PI34911 | USA |  | Uncertain |
| PI35038 | SUMAC | PI35038 | USA |  | Uncertain |
| PI365512 | 1130 | PI365512 | SAF | Caudatum | Cultivar |
| PI365512_dup |  | PI365512_dup | SAF | Caudatum | Cultivar |
| PI369484 | 26 | PI369484 | WAF | Wild | Uncertain |
| PI369487 | 1 | PI369487 | WAF | Wild | Uncertain |
| PI383856 | AF-28 | PI383856 | LAC | Caudatum | Uncertain |
| PI452542 | ETS 2029 | PI452542 | EAF | Durra | Cultivar |
| PI452544 | ETS 2030 | PI452544 | EAF | Durra | Cultivar |
| PI452619 | ETS 2069 | PI452619 | EAF | Guinea | Cultivar |
| PI452692 | ETS 2110 | PI452692 | EAF | Durra | Cultivar |
| PI453106 |  | PI453106 | EAF | Durra | Cultivar |
| PI453177 |  | PI453177 | EAF | Durra | Cultivar |
| PI453696 |  | PI453696 | EAF | Durra | Cultivar |
| PI455217 | ETS 3455 | PI455217 | EAF | Durra | Cultivar |
| PI455280 |  | PI455280 | EAF | Durra | Cultivar |
| PI455301 | ETS 3493 | PI455301 | EAF | Kafir | Cultivar |
| PI455307_dup |  | PI455307_dup | EAF | Durra | Uncertain |
| PI474825 |  | PI474825 | SEEU_ME | Durra | Cultivar |
| PI48770 | WHITE KAFIR | PI48770 | SAF |  | Breeding |
| PI505705 | ZM/A 5204 | PI505705 | EAF | Kafir | Landrace |
| PI506030 | Tchinlouol | PI506030 | WAF | Guinea | Cultivar |
| PI511355 | SMITH | PI511355 | USA |  | Cultivar |
| PI513898 | Abokoun Wewe | PI513898 | WAF | Guinea | Cultivar |
| PI514456 | IS 20043 | PI514456 | WAF | Guinea | Cultivar |
| PI521019 | Andiwo | PI521019 | EAF | Durra | Cultivar |
| PI521152 |  | PI521152 | EAF | Caudatum | Cultivar |
| PI521280 | Muya | PI521280 | EAF | Durra | Cultivar |
| PI521281 | Mwee | PI521281 | EAF | Bicolor | Cultivar |
| PI521290 | Bishehe | PI521290 | EAF | Guinea | Cultivar |
| PI522160 | Bachara | PI522160 | WAF | Complex | Uncertain |
| PI524475 | Nvarkan | PI524475 | NAF | Caudatum | Cultivar |
| PI524718 | Yabia | PI524718 | NAF | Wild | Uncertain |
| PI524948 | Namisankele | PI524948 | EAF | Kafir | Uncertain |
| PI524948_dup | Namisankele_dup | PI524948_dup | EAF | Kafir | Uncertain |
| PI525049 |  | PI525049 | EAF | Guinea | Cultivar |
| PI525059 | MW 719 | PI525059 | EAF | Caudatum | Uncertain |
| PI52606 |  | PI52606 | SAF | Kafir | Cultivar |
| PI526905 | AMM 721 | PI526905 | EAF | Caudatum | Cultivar |
| PI527045 | AMM 1051 | PI527045 | EAF | Caudatum | Cultivar |
| PI532564 | TCD 018 | PI532564 | NAF | Wild | Wild |
| PI532565 | TCD 050 | PI532565 | NAF | Wild | Wild |
| PI532566 | TCD 070 | PI532566 | NAF | Wild | Wild |
| PI532568 | TCD 156 | PI532568 | NAF | Wild | Wild |
| PI533750 | K.3 Perimanjial Irungu Cholam | PI533750 | SAS | Bicolor | Breeding |
| PI533752 | 54.K.94(Witchweed res.) | PI533752 | SAF | Caudatum | Breeding |
| PI533754 | SAP-219 | PI533754 | NAF | Bicolor | Breeding |
| PI533755 | Feterita Gondal | PI533755 | NAF | Caudatum | Breeding |
| PI533757 | Monshal | PI533757 | NAF | Kafir | Breeding |
| PI533758 | IS 18684 | PI533758 | NAF | Caudatum | Breeding |
| PI533759 | Mugbash 56/56 | PI533759 | NAF | Caudatum | Breeding |
| PI533760 | Zera Zera White | PI533760 | NAF | Caudatum | Breeding |
| PI533761 | SAP-230 | PI533761 | EAF | Caudatum | Breeding |
| PI533762 | Tambroro 7 | PI533762 | NAF | Durra | Breeding |
| PI533766 | No. 4 Hadoui | PI533766 | WAF | Guinea | Breeding |
| PI533769 | 290 Feterita Shendi 2 | PI533769 | NAF | Kafir | Breeding |
| PI533776 | KA 3 | PI533776 | WAF | Caudatum | Breeding |
| PI533785 | SO 16 | PI533785 | USA | Guinea | Breeding |
| PI533788 | SAP-263 | PI533788 | NAF | Durra | Breeding |
| PI533789 | Kodilib | PI533789 | NAF | Guinea | Breeding |
| PI533792 | No. 1 Gambela | PI533792 | EAF | Caudatum | Breeding |
| PI533794 | No. 5 Gambela | PI533794 | EAF | Caudatum | Breeding |
| PI533799 | SAP-404 | PI533799 | EAF | Caudatum | Breeding |
| PI533800 | IS 12666C | PI533800 | EAF | Caudatum | Breeding |
| PI533807 | SAP-206 | PI533807 | WAF | Kafir | Breeding |
| PI533810 | Karad 2-7-11 | PI533810 | SAS | Durra | Breeding |
| PI533814 | N.9 Cherukupatcha Jonna | PI533814 | SAS | Durra | Breeding |
| PI533821 | AS4660 Kikuma | PI533821 | EAF | Caudatum | Breeding |
| PI533822 | AS4616 Balangira-Mwanza | PI533822 | EAF | Kafir | Breeding |
| PI533824 | Bonkum | PI533824 | WAF | Durra | Breeding |
| PI533830 | Culum Brick | PI533830 | NAF | Caudatum | Breeding |
| PI533831 | Deburr | PI533831 | NAF | Kafir | Breeding |
| PI533833 | Sorghum Soroti | PI533833 | EAF | Caudatum | Breeding |
| PI533838 | BA45 Faria Bonkum | PI533838 | WAF | Caudatum | Breeding |
| PI533839 | K037 Camjin | PI533839 | WAF | Guinea | Breeding |
| PI533841 | ZA41 Danye | PI533841 | WAF | Guinea | Breeding |
| PI533842 | Nandyal | PI533842 | SAS | Durra | Breeding |
| PI533843 | SV 34 | PI533843 | SAS | Guinea | Breeding |
| PI533852 | Cholia Talijhari | PI533852 | SAS | Durra | Breeding |
| PI533855 | Chanan Singoo | PI533855 | SAS | Guinea | Breeding |
| PI533856 | Jola Nandyal | PI533856 | SAS | Durra | Breeding |
| PI533863 | 740 Oua Berr | PI533863 | NAF | Kafir | Breeding |
| PI533866 | 255 Tirter | PI533866 |  |  | Breeding |
| PI533869 | Msumbji SB 117 | PI533869 | EAF | Guinea | Breeding |
| PI533871 | M 1 | PI533871 | WAF | Caudatum | Breeding |
| PI533876 | KA 12 Janjari | PI533876 | WAF | Caudatum | Breeding |
| PI533877 | KA 15 Yazgar Giwa | PI533877 | WAF | Caudatum | Breeding |
| PI533878 | KA 21 Gajerar Kaura | PI533878 | WAF | Caudatum | Breeding |
| PI533882 | KO 61 Baragwan Doki | PI533882 | WAF | Caudatum | Breeding |
| PI533901 | A-96 | PI533901 | EAS | Caudatum | Breeding |
| PI533902 | Orange No. l, Baijo | PI533902 | EAF | Durra | Breeding |
| PI533903 | No. 35 Mab | PI533903 | EAF | Bicolor | Breeding |
| PI533910 | Klor | PI533910 | NAF | Caudatum | Breeding |
| PI533911 | Kokla | PI533911 | NAF | Caudatum | Breeding |
| PI533912 | Mendo | PI533912 | NAF |  | Breeding |
| PI533913 | Nyan Dok | PI533913 | NAF | Guinea | Breeding |
| PI533915 | No. 755 Muzeba | PI533915 | EAF |  | Breeding |
| PI533919 | R1_4 | PI533919 | EAF | Durra | Breeding |
| PI533921 | R1_38 | PI533921 | EAF | Durra | Breeding |
| PI533924 | R3_121 | PI533924 | EAF | Durra | Breeding |
| PI533927 | J.A.T.S. #67 | PI533927 | EAF | Bicolor | Breeding |
| PI533936 | 1903 AS 4633 | PI533936 | EAF | Guinea | Breeding |
| PI533937 | AS 4136 Masaka Luwemba | PI533937 | SAF | Kafir | Breeding |
| PI533938 | AS 5826 Holcus | PI533938 | SAF | Caudatum | Breeding |
| PI533939 | AS 4055 N Kambwa | PI533939 | EAF | Caudatum | Breeding |
| PI533940 | AS 4601 Pawaga | PI533940 | EAF | Bicolor | Breeding |
| PI533943 | AS 2613 NT | PI533943 | SAS | Durra | Breeding |
| PI533948 | Nebraska 6350 | PI533948 | USA | Guinea | Breeding |
| PI533949 | Maja Abiad Q2/2/68 | PI533949 | NAF | Kafir | Breeding |
| PI533954 | P 3742 | PI533954 | SAF | Caudatum | Breeding |
| PI533955 | P 3749 Q2/5/62 | PI533955 | SAF | Kafir | Breeding |
| PI533956 | MN 586 (preconverted) | PI533956 | SAF | Durra | Breeding |
| PI533957 | MN 707 (preconverted) | PI533957 |  | Caudatum | Breeding |
| PI533961 | Wit Lichtenburg DL/59/1530 | PI533961 | SAF | Caudatum | Breeding |
| PI533962 | Malwal Aweil | PI533962 | NAF | Caudatum | Breeding |
| PI533964 | Safara Kordafan | PI533964 | NAF | Caudatum | Breeding |
| PI533965 | SAP-148 | PI533965 | EAF | Caudatum | Breeding |
| PI533967 | SB-283 | PI533967 | EAF | Caudatum | Breeding |
| PI533970 | 2033Z-3 | PI533970 | EAF | Caudatum | Breeding |
| PI533972 | Dobbs | PI533972 | EAF | Caudatum | Breeding |
| PI533976 | Framiola DL/59/1539 | PI533976 | SAF | Kafir | Breeding |
| PI533979 | Bulfontein White Kafir Corn DL/60/133 | PI533979 | SAF | Kafir | Breeding |
| PI533980 | SAP-275 | PI533980 | EAS | Kafir | Breeding |
| PI533985 | Barking 119 | PI533985 | NAF | Caudatum | Breeding |
| PI533986 | Huria White 621 | PI533986 | NAF | Caudatum | Breeding |
| PI533987 | Kireniga 317 | PI533987 | NAF | Caudatum | Breeding |
| PI533989 | Nyithin | PI533989 | NAF | Caudatum | Breeding |
| PI533991 | Sinidyil 177 | PI533991 | NAF | Guinea | Breeding |
| PI533996 | Hamaisi 38 | PI533996 | NAF | Durra | Breeding |
| PI533997 | MN 1592 (preconverted) | PI533997 | SEEU_ME | Guinea | Breeding |
| PI533998 | Brawley | PI533998 |  |  | Breeding |
| PI534009 | Yerra Jonna Goda | PI534009 | SAS | Durra | Breeding |
| PI534021 | Jola Nandyal | PI534021 | SAS | Durra | Breeding |
| PI534028 | Jonar Tamargundi | PI534028 | SAS | Durra | Breeding |
| PI534037 | 6428 Sian | PI534037 | NAF | Guinea | Breeding |
| PI534046 | 420 Kalatilansa | PI534046 | NAF | Guinea | Breeding |
| PI534047 | 450 Bori Light Brown | PI534047 | NAF | Caudatum | Breeding |
| PI534053 | T 28 | PI534053 | EAF | Caudatum | Breeding |
| PI534054 | 194 Kano | PI534054 | EAF | Kafir | Breeding |
| PI534063 |  | PI534063 | WAF | Guinea | Breeding |
| PI534070 | BE 25 | PI534070 | WAF | Guinea | Breeding |
| PI534075 | KA 24 | PI534075 | WAF | Caudatum | Breeding |
| PI534079 | PL 47 | PI534079 | WAF | Caudatum | Breeding |
| PI534088 | ZA 6 | PI534088 | WAF | Caudatum | Breeding |
| PI534092 | ZA 71 | PI534092 | WAF | Caudatum | Breeding |
| PI534096 | SO 85 | PI534096 | WAF | Guinea | Breeding |
| PI534097 | HC 6028 | PI534097 | EAS | Kafir | Breeding |
| PI534101 | A-106 | PI534101 | EAS | Caudatum | Breeding |
| PI534105 | EC 21361 G30 | PI534105 | EAF | Kafir | Breeding |
| PI534108 | EC 21428 SB 63 | PI534108 | EAF | Kafir | Breeding |
| PI534112 | EC 21463 STR 5/1 | PI534112 | SAS | Caudatum | Breeding |
| PI534114 | Accho Karuho | PI534114 | SAS | Caudatum | Breeding |
| PI534115 | Akwu | PI534115 | EAF | Caudatum | Breeding |
| PI534116 | Mashica | PI534116 | EAF | Durra | Breeding |
| PI534120 | Sumac No. 2, Baijo, Shoa, Ethiopia | PI534120 | EAF |  | Breeding |
| PI534123 | No. 25 Gobo Kaichama Ethiopia | PI534123 | EAF | Durra | Breeding |
| PI534124 | No. 28 Lyle Marawa Ethiopia | PI534124 | EAF | Guinea | Breeding |
| PI534128 | No. 65 Amelsie Kimbolcha Ethiopia | PI534128 | EAF | Durra | Breeding |
| PI534132 | No. 59 Bekedjie Kembolcha Ethiopia | PI534132 | EAF | Durra | Breeding |
| PI534133 | No. 69 Mashela Tinguish Warakul Ethiopi | PI534133 | EAF | Durra | Breeding |
| PI534135 | No. 64 Netch Addesho Ethiopia | PI534135 | EAF | Durra | Breeding |
| PI534137 | Budy | PI534137 | NAF | Caudatum | Breeding |
| PI534138 | Matchikah | PI534138 | NAF | Guinea | Breeding |
| PI534139 | Tuery 11 | PI534139 | NAF |  | Breeding |
| PI534144 | Kabutuwa | PI534144 | EAF | Durra | Breeding |
| PI534145 | No. 902 Sorghum arundi | PI534145 | SAF |  | Breeding |
| PI534148 | R1_21 | PI534148 | EAF | Durra | Breeding |
| PI534155 | R3_80 | PI534155 | EAF | Durra | Breeding |
| PI534157 | R4_B1 | PI534157 | EAF | Caudatum | Breeding |
| PI534163 | Rexx | PI534163 | USA | Caudatum | Breeding |
| PI534165 | Kalati Consa | PI534165 | NAF |  | Breeding |
| PI534167 | F.R. Miller | PI534167 |  | Durra | Breeding |
| PI535783 | N98 | PI535783 | USA |  | Breeding |
| PI535783_dup |  | PI535783_dup | USA |  | Breeding |
| PI535785 | N100 | PI535785 | USA |  | Breeding |
| PI535792 | N107 | PI535792 | USA |  | Breeding |
| PI535793 | N108 | PI535793 | USA |  | Breeding |
| PI535793_dup |  | PI535793_dup | USA |  | Breeding |
| PI535794 | N109 | PI535794 | USA |  | Breeding |
| PI535795 | N110 | PI535795 | USA |  | Breeding |
| PI535796 | N111 | PI535796 | USA |  | Breeding |
| PI535995 | C120 | PI535995 | SAF | Wild | Wild |
| PI540518 | SEGAOLANE | PI540518 | SAF |  | Cultivar |
| PI540816 | Tx2882 | PI540816 | USA |  | Breeding |
| PI542406 | R LINE AR2002 | PI542406 | USA |  | Breeding |
| PI542718 | SAN CHI SAN | PI542718 | EAS | Bicolor | Breeding |
| PI548797 | Tx2891 | PI548797 | USA |  | Breeding |
| PI550604 | ZERNOGRAPSKOE 100 | PI550604 | SEEU_ME | Caudatum | Breeding |
| PI552851 | 7035S | PI552851 | USA |  | Breeding |
| PI552856 | A/BTx626 | PI552856 | USA |  | Breeding |
| PI552861 | A/BTx631 | PI552861 | USA |  | Breeding |
| PI561071 | RTx436 | PI561071 | USA |  | Breeding |
| PI561072 | A/BTxARG-1 | PI561072 | USA |  | Breeding |
| PI561073 | A/BTx635 | PI561073 | USA |  | Breeding |
| PI561472 | SURENO | PI561472 | LAC |  | Cultivar |
| PI562715 | KAURA | PI562715 | WAF |  | Cultivar |
| PI562717 | PERENNIAL SWEET SUDAN | PI562717 | USA | Bicolor | Cultivar |
| PI562730 | GRAIN GRASS 3A | PI562730 |  |  | Cultivar |
| PI562732 | LGV 88-1 (DWARF) | PI562732 | LAC | Durra | Cultivar |
| PI562781 | IS 3869 | PI562781 | WAF | Guinea | Cultivated |
| PI562897 | FARAKO-BA | PI562897 | WAF | Guinea | Cultivar |
| PI562897_dup |  | PI562897_dup | WAF | Guinea | Cultivar |
| PI562963 | BUZARI | PI562963 | WAF | Caudatum | Cultivar |
| PI562970 | FARAFARA | PI562970 | WAF | Guinea | Cultivar |
| PI562971 | FARAFARA | PI562971 | WAF | Guinea | Cultivar |
| PI562971_dup |  | PI562971_dup | WAF | Guinea | Cultivar |
| PI562981 | EKA OR OKA BABA | PI562981 | WAF | Guinea | Cultivar |
| PI562982 | EKA OR OKA BABA | PI562982 | WAF | Guinea | Cultivar |
| PI562990 | MURBAN GANGAU | PI562990 | WAF | Guinea | Cultivar |
| PI562991 | GIWA | PI562991 | WAF | Guinea | Cultivar |
| PI562991_dup |  | PI562991_dup | WAF | Guinea | Cultivar |
| PI562994 |  | PI562994 | WAF | Guinea | Cultivar |
| PI562997 | EKPAN DZUNGI | PI562997 | WAF | Guinea | Cultivar |
| PI562998 | MASUNGI | PI562998 | WAF | Guinea | Cultivar |
| PI563002 | EKPAN DZURUGI | PI563002 | WAF | Guinea | Cultivar |
| PI563006 | GAGAYA | PI563006 | WAF | Guinea | Cultivar |
| PI563009 | IS 7739 | PI563009 | WAF | Guinea | Cultivated |
| PI563020 | OKA FUNFUN | PI563020 | WAF | Guinea | Cultivar |
| PI563021 | TAPASOHIRA | PI563021 | WAF | Guinea | Cultivar |
| PI563022 | ESSTENE | PI563022 | WAF | Guinea | Cultivar |
| PI563023 | GIWA KANBA FARIN KONA | PI563023 | WAF | Guinea | Cultivar |
| PI563032 | COLLIER 706C | PI563032 | EAS | Bicolor | Cultivar |
| PI563196 | IS 9128 | PI563196 | EAF | Guinea | Cultivated |
| PI563329 |  | PI563329 | EAF | Durra | Cultivated |
| PI563332 |  | PI563332 | EAF | Durra | Cultivated |
| PI563338 |  | PI563338 | WAF | Guinea | Cultivated |
| PI563348 |  | PI563348 | WAF | Guinea | Cultivated |
| PI563350 |  | PI563350 | WAF | Guinea | Cultivated |
| PI563355 | BOLOUNGOU | PI563355 | WAF | Guinea | Cultivar |
| PI563509 | IS 12685 | PI563509 | EAF | Durra | Cultivated |
| PI564163 | BTx623 | PI564163 | USA |  | Breeding |
| PI564164 | RTx433 | PI564164 | USA |  | Breeding |
| PI564165 | RTx434 | PI564165 | USA |  | Breeding |
| PI565121 | MACIA | PI565121 | EAF |  | Cultivar |
| PI566819 | DELLA | PI566819 | USA |  | Cultivar |
| PI568717 |  | PI568717 | SAF | Durra | Cultivated |
| PI569097 |  | PI569097 | NAF | Durra | Cultivated |
| PI569148 |  | PI569148 | NAF | Durra | Cultivated |
| PI569244 |  | PI569244 | NAF | Durra | Cultivated |
| PI569264 |  | PI569264 | NAF | Caudatum | Cultivated |
| PI569416 |  | PI569416 | NAF | Caudatum | Cultivated |
| PI569418 |  | PI569418 | NAF | Guinea | Cultivated |
| PI569419 |  | PI569419 | NAF | Caudatum | Cultivated |
| PI569420 |  | PI569420 | NAF | Guinea | Cultivated |
| PI569422 |  | PI569422 | NAF | Caudatum | Cultivated |
| PI569423 |  | PI569423 | NAF | Guinea | Cultivated |
| PI569425 |  | PI569425 | NAF | Caudatum | Cultivated |
| PI569427 |  | PI569427 | NAF | Caudatum | Cultivated |
| PI569433 |  | PI569433 | NAF | Caudatum | Cultivated |
| PI569435 |  | PI569435 | NAF | Guinea | Cultivated |
| PI569443 |  | PI569443 | NAF | Kafir | Cultivated |
| PI569445 |  | PI569445 | NAF | Guinea | Cultivated |
| PI569446 |  | PI569446 | NAF | Guinea | Cultivated |
| PI569447 |  | PI569447 | NAF | Caudatum | Cultivated |
| PI569453 |  | PI569453 | NAF | Guinea | Cultivated |
| PI569454 |  | PI569454 | NAF | Guinea | Cultivated |
| PI569455 |  | PI569455 | NAF | Guinea | Cultivated |
| PI569457 |  | PI569457 | NAF | Caudatum | Cultivated |
| PI569458 |  | PI569458 | NAF | Caudatum | Cultivated |
| PI569459 |  | PI569459 | NAF | Caudatum | Cultivated |
| PI569460 |  | PI569460 | NAF | Caudatum | Cultivated |
| PI569465 |  | PI569465 | NAF | Caudatum | Cultivated |
| PI569886 |  | PI569886 | NAF | Guinea | Cultivated |
| PI570031 |  | PI570031 | NAF | Guinea | Cultivated |
| PI570038_redo |  | PI570038_redo | NAF | Kafir | Cultivated |
| PI570039_redo |  | PI570039_redo | NAF | Guinea | Cultivated |
| PI570042 |  | PI570042 | NAF | Guinea | Cultivated |
| PI570053 |  | PI570053 | NAF | Caudatum | Cultivated |
| PI570071 |  | PI570071 | NAF | Guinea | Cultivated |
| PI570073 | IS 22851 | PI570073 | NAF | Guinea | Cultivated |
| PI570074 | IS 22852 | PI570074 | NAF | Guinea | Cultivated |
| PI570075 |  | PI570075 | NAF | Guinea | Cultivated |
| PI570076 | IS 22854 | PI570076 | NAF | Guinea | Cultivated |
| PI570085 | IS 22863 | PI570085 | NAF | Guinea | Cultivated |
| PI570087 | IS 22865 | PI570087 | NAF | Guinea | Cultivated |
| PI570090 | IS 22868 | PI570090 | NAF | Guinea | Cultivated |
| PI570091 | IS 22869 | PI570091 | NAF | Guinea | Cultivated |
| PI570096 | IS 22874 | PI570096 | NAF | Guinea | Cultivated |
| PI570106 | IS 22884 | PI570106 | NAF | Guinea | Cultivated |
| PI570110 | IS 22888 | PI570110 | NAF | Guinea | Cultivated |
| PI570110_dup |  | PI570110_dup | NAF | Guinea | Cultivated |
| PI570114 | IS 22892 | PI570114 | NAF | Guinea | Cultivated |
| PI570145 | IS 22923 | PI570145 | NAF | Guinea | Cultivated |
| PI570371 | IS 25045 | PI570371 | NAF | Guinea | Cultivated |
| PI570373 |  | PI570373 | NAF | Guinea | Cultivated |
| PI570388 |  | PI570388 | NAF | Guinea | Cultivated |
| PI570393 | IS 26849 | PI570393 | NAF | Caudatum | Cultivated |
| PI570400 | LABALWA | PI570400 | NAF | Guinea | Cultivated |
| PI570431 |  | PI570431 | NAF | Durra | Cultivated |
| PI570712 | El Safra | PI570712 | NAF | Caudatum | Uncertain |
| PI570813 | Dwarf Shallu | PI570813 | NAF | Durra | Uncertain |
| PI570916 | Makai | PI570916 | NAF | Caudatum | Uncertain |
| PI570928 | Birgalli Ahmar | PI570928 | NAF | Caudatum | Uncertain |
| PI570969 | Feterita Shendi 2 | PI570969 | NAF | Caudatum | Uncertain |
| PI571001 | Tabrere | PI571001 | NAF | Caudatum | Uncertain |
| PI571020 | Feterita Abu Direiera | PI571020 | NAF | Durra | Uncertain |
| PI571022 | Colum 2 | PI571022 | NAF | Caudatum | Uncertain |
| PI573193 | A/7 | PI573193 | NAF | Caudatum | Cultivated |
| PI576130 | ICSV 745 | PI576130 | SAS |  | Breeding |
| PI576332 | SAP-287 | PI576332 | EAS | Bicolor | Breeding |
| PI576333 | SAP-306 | PI576333 | USA | Guinea | Breeding |
| PI576337 | SAP-280 | PI576337 | EAF | Wild | Breeding |
| PI576339 | SAP-312 | PI576339 | EAF | Kafir | Breeding |
| PI576340 | Nkuli Swaziland | PI576340 | SAF | Kafir | Breeding |
| PI576345 | SAP-294 | PI576345 | SAF | Kafir | Breeding |
| PI576347 | SAP-342 | PI576347 | USA | Bicolor | Breeding |
| PI576348 | SAP-341 | PI576348 | USA | Bicolor | Breeding |
| PI576349 | SAP-343 | PI576349 | USA | Bicolor | Breeding |
| PI576350 | SAP-417 | PI576350 | USA | Kafir | Breeding |
| PI576352 | Marupantse | PI576352 | SAF | Kafir | Breeding |
| PI576359 | Butivori | PI576359 | SAS | Guinea | Breeding |
| PI576364 | Chari Uri | PI576364 | SAS | Caudatum | Breeding |
| PI576366 | Jowar Red Jankinagar | PI576366 | SAS | Durra | Breeding |
| PI576373 | SAP-325 | PI576373 | EAS | Caudatum | Breeding |
| PI576375 | SAP-124 | PI576375 | EAF | Durra | Breeding |
| PI576376 | SAP-125 | PI576376 | EAF | Durra | Breeding |
| PI576380 | Mashica | PI576380 | EAF | Caudatum | Breeding |
| PI576381 | Mashica | PI576381 | EAF | Durra | Breeding |
| PI576385 | SAP-135 | PI576385 | WAF |  | Breeding |
| PI576386 | SAP-347 | PI576386 | EAF |  | Breeding |
| PI576387 | Awanlek | PI576387 | NAF |  | Breeding |
| PI576390 | Kharuth Waragel | PI576390 | SAS | Durra | Breeding |
| PI576391 | K.1 Irungu cholam | PI576391 | SAS | Bicolor | Breeding |
| PI576393 | MN 708 (preconverted) | PI576393 | EAF | Caudatum | Breeding |
| PI576394 | Lambas | PI576394 | NAF | Caudatum | Breeding |
| PI576396 | SAP-225 | PI576396 | EAF | Caudatum | Breeding |
| PI576399 | IS 3515C | PI576399 | NAF | Caudatum | Breeding |
| PI576401 | Mehra Sonthia | PI576401 | SAS | Durra | Breeding |
| PI576418 | SAP-147 | PI576418 | WAF | Guinea | Breeding |
| PI576422 | SAP-141 | PI576422 | SAF | Kafir | Breeding |
| PI576425 | SAP-150 | PI576425 | EAF | Durra | Breeding |
| PI576426 | SAP-128 | PI576426 | EAF | Durra | Breeding |
| PI576428 | Mashica | PI576428 | EAF | Caudatum | Breeding |
| PI576434 | Ex-Mubi | PI576434 | WAF | Kafir | Breeding |
| PI576435 | Lula | PI576435 | EAF | Kafir | Breeding |
| PI576437 | SAP-155 | PI576437 | LAC |  | Breeding |
| PI583832 | TOP 76-6 | PI583832 | USA |  | Cultivar |
| PI585291 | Tx2903 | PI585291 | USA |  | Breeding |
| PI585295 | Tx2907 | PI585295 | USA |  | Breeding |
| PI585406 | IS 24800 | PI585406 | WAF | Guinea | Cultivated |
| PI585448 | IS 25050 | PI585448 | WAF | Guinea | Cultivated |
| PI585452 | IS 25059 | PI585452 | WAF | Guinea | Cultivated |
| PI585454 | IS 25061 | PI585454 | WAF | Guinea | Cultivated |
| PI585461 | IS 25086 | PI585461 | WAF | Guinea | Cultivated |
| PI585467 | IS 25108 | PI585467 | WAF | Guinea | Cultivated |
| PI585577 | IS 25514 | PI585577 | EAF | Durra | Cultivated |
| PI585608 | GIKONDO | PI585608 | EAF | Caudatum | Cultivated |
| PI585961 | IS 26386 | PI585961 | WAF | Guinea | Cultivated |
| PI585966 | DJOFELA | PI585966 | WAF | Guinea | Cultivated |
| PI585966_dup |  | PI585966_dup | WAF | Guinea | Cultivated |
| PI586435_redo |  | PI586435_redo | SEEU_ME | Guinea | Cultivated |
| PI586499 |  | PI586499 | SAF | Kafir | Cultivated |
| PI586541 | TRACY | PI586541 | AUS | Caudatum | Cultivar |
| PI586541_dup |  | PI586541_dup | AUS | Caudatum | Cultivar |
| PI586792 | Tarbroro White | PI586792 | NAF | Caudatum | Uncertain |
| PI593916 | B3SC599-6-9188 | PI593916 | USA |  | Breeding |
| PI595699 | SAP-340 | PI595699 | USA | Durra | Breeding |
| PI595702 | SAP-311 | PI595702 | USA | Kafir | Breeding |
| PI595714 | SAP-139 | PI595714 | USA | Caudatum | Breeding |
| PI595718 | SAP-162 | PI595718 | USA | Kafir | Breeding |
| PI595720 | SAP-149 | PI595720 | USA | Durra | Breeding |
| PI595739 | SAP-131 | PI595739 | USA | Caudatum | Breeding |
| PI595740 | SAP-133 | PI595740 | USA | Caudatum | Breeding |
| PI595741 | SAP-134 | PI595741 | USA | Guinea | Breeding |
| PI595743 | SAP-154 | PI595743 | USA | Guinea | Breeding |
| PI595744 | SAP-157 | PI595744 | USA | Kafir | Breeding |
| PI595745 | SAP-159 | PI595745 | USA |  | Breeding |
| PI597945 | SAP-336 | PI597945 | USA | Durra | Breeding |
| PI597946 | SAP-286 | PI597946 | USA | Guinea | Breeding |
| PI597949 | SAP-146 | PI597949 | USA | Guinea | Breeding |
| PI597950 | SAP-250 | PI597950 | USA | Guinea | Breeding |
| PI597951 | SAP-264 | PI597951 | USA | Guinea | Breeding |
| PI597952 | SAP-323 | PI597952 | USA | Caudatum | Breeding |
| PI597957 | SAP-151 | PI597957 | USA | Durra | Breeding |
| PI597958 | SAP-197 | PI597958 | USA | Kafir | Breeding |
| PI597960 | SAP-137 | PI597960 | USA | Caudatum | Breeding |
| PI597961 | SAP-138 | PI597961 | USA |  | Breeding |
| PI597964 | SAP-166 | PI597964 | USA | Caudatum | Breeding |
| PI597965 | SAP-395 | PI597965 | USA | Caudatum | Breeding |
| PI597967 | SAP-167 | PI597967 | USA |  | Breeding |
| PI597968 | SAP-168 | PI597968 | USA | Guinea | Breeding |
| PI597971 | SAP-170 | PI597971 | USA | Caudatum | Breeding |
| PI597972 | SAP-398 | PI597972 | USA | Durra | Breeding |
| PI597973 | SAP-171 | PI597973 | USA | Durra | Breeding |
| PI597976 | SAP-172 | PI597976 | USA | Guinea | Breeding |
| PI597980 | SAP-173 | PI597980 | USA | Caudatum | Breeding |
| PI597982 | SAP-175 | PI597982 | USA | Caudatum | Breeding |
| PI598069 | RTx2909 | PI598069 | USA |  | Breeding |
| PI598070 | RTx2910 | PI598070 | USA |  | Breeding |
| PI601816 | ICSV 400 | PI601816 | WAF |  | Cultivar |
| PI606706 |  | PI606706 | USA |  | Breeding |
| PI607931 | Tx2911 | PI607931 | USA |  | Breeding |
| PI609456 | SAP-386 | PI609456 | WAF |  | Uncertain |
| PI609612 | HAMBA BIBA | PI609612 | WAF | Bicolor | Cultivated |
| PI613536 | KS 115 | PI613536 | USA |  | Breeding |
| PI619807 | IS 31047 | PI619807 | EAF |  | Landrace |
| PI619838 | IS 31083 | PI619838 | EAF |  | Cultivar |
| PI620072 | IS 31384 | PI620072 | EAF |  | Landrace |
| PI620157_redo |  | PI620157_redo | EAF |  | Landrace |
| PI629034 | RTx437 | PI629034 | USA |  | Breeding |
| PI629040 | Tx2917 | PI629040 | USA |  | Breeding |
| PI629059 | BTx2928 | PI629059 | USA | Kafir | Breeding |
| PI63715_redo |  | PI63715_redo | EAS | Kafir | Uncertain |
| PI641810 |  | PI641810 |  |  | Cultivar |
| PI641815 |  | PI641815 |  |  | Cultivar |
| PI641817 |  | PI641817 |  |  | Cultivar |
| PI641821 |  | PI641821 |  |  | Cultivar |
| PI641824 | KANSAS ORANGE | PI641824 |  |  | Breeding |
| PI641829 |  | PI641829 |  |  | Cultivar |
| PI641830 |  | PI641830 |  |  | Cultivar |
| PI641834 | PLANTER | PI641834 |  |  | Cultivar |
| PI641835 |  | PI641835 |  |  | Cultivar |
| PI641836 | ROX ORANGE | PI641836 |  |  | Breeding |
| PI641849 | WACONIA | PI641849 |  |  | Uncertain |
| PI641850 |  | PI641850 |  |  | Cultivar |
| PI641860 |  | PI641860 |  |  | Cultivar |
| PI641862 |  | PI641862 |  |  | Cultivar |
| PI641874 | DAY MILO | PI641874 |  |  | Breeding |
| PI641892 |  | PI641892 |  |  | Cultivar |
| PI641909 |  | PI641909 | NAF |  | Cultivar |
| PI642791 | BTx640 | PI642791 | USA |  | Breeding |
| PI642793 | BTx641 | PI642793 | USA |  | Breeding |
| PI642992 | Standard Early Hegari | PI642992 |  |  | Breeding |
| PI642998 | Black Spanish | PI642998 |  |  | Uncertain |
| PI643008_redo |  | PI643008_redo |  |  | Uncertain |
| PI643016_redo |  | PI643016_redo |  |  | Uncertain |
| PI646242 |  | PI646242 | EAF |  | Cultivar |
| PI646251 |  | PI646251 | EAF |  | Cultivar |
| PI646266_redo |  | PI646266_redo | EAF |  | Uncertain |
| PI647743 | Dawa U.A.R. busari | PI647743 | NAF | Durra | Uncertain |
| PI647745 | IS 6710 | PI647745 | WAF | Durra | Uncertain |
| PI651102 | IS 1201 | PI651102 | WAF | Kafir | Uncertain |
| PI651113 | IS 7271 | PI651113 | WAF | Guinea | Uncertain |
| PI651116 | IS 7361 | PI651116 | WAF | Guinea | Uncertain |
| PI651124 | IS 7416 | PI651124 | WAF | Guinea | Uncertain |
| PI651131 | IS 7633 | PI651131 | WAF | Guinea | Uncertain |
| PI651141 | Jana dawa | PI651141 | WAF | Guinea | Uncertain |
| PI651167 | IS 7273 | PI651167 | WAF | Guinea | Uncertain |
| PI651168 | IS 7274 | PI651168 | WAF | Durra | Uncertain |
| PI651178 | IS 7735 | PI651178 | WAF | Guinea | Uncertain |
| PI651187 | IS 6392 | PI651187 | SAS | Guinea | Uncertain |
| PI651491 |  | PI651491 | USA |  | Cultivar |
| PI651492 | COWLEY | PI651492 | USA |  | Uncertain |
| PI651493 | RAMADA | PI651493 | USA |  | Cultivar |
| PI651495 |  | PI651495 | USA |  | Cultivar |
| PI651496 | RIO | PI651496 |  |  | Cultivar |
| PI651497 |  | PI651497 | USA |  | Cultivar |
| PI653411 |  | PI653411 | USA |  | Cultivar |
| PI653616 | WRAY | PI653616 | USA |  | Cultivar |
| PI653616_dup | WRAY_dup | PI653616_dup | USA |  | Cultivar |
| PI653617 | KELLER | PI653617 | USA |  | Cultivar |
| PI655970 | Standard Blackhull Kafir | PI655970 | USA |  | Breeding |
| PI655971 | STANDARD WHITE MILO | PI655971 |  |  | Breeding |
| PI655972 | PINK KAFIR | PI655972 | USA |  | Breeding |
| PI655973 | SPUR FETERITA | PI655973 | USA |  | Breeding |
| PI655974 | TEXAS BLACKHULL KAFIR | PI655974 | USA |  | Breeding |
| PI655975 | WHEATLAND | PI655975 | USA |  | Breeding |
| PI655976 | RED KAFIR | PI655976 | USA |  | Breeding |
| PI655977 | TAM2566 | PI655977 | USA |  | Breeding |
| PI655978 | Tx2737 | PI655978 | USA |  | Breeding |
| PI655979 | Tx2741 | PI655979 | USA |  | Breeding |
| PI655980 | Tx2785 | PI655980 | USA |  | Breeding |
| PI655981 | 87C424 | PI655981 | WAF |  | Cultivar |
| PI655982 | SAP-380 | PI655982 | AUS |  | Uncertain |
| PI655983 | SUGAR DRIP | PI655983 |  |  | Breeding |
| PI655984 | CHILTEX | PI655984 | USA |  | Breeding |
| PI655985 | PLAINSMAN | PI655985 | USA |  | Breeding |
| PI655986 | CAPROCK | PI655986 | USA |  | Breeding |
| PI655987 | MARTIN | PI655987 | USA |  | Breeding |
| PI655988 | COMBINE KAFIR-60 | PI655988 | USA |  | Breeding |
| PI655989 | REDBINE-60 | PI655989 | USA |  | Breeding |
| PI655990 | COMBINE 7078 | PI655990 | USA |  | Breeding |
| PI655991 | Tx378 (REDLAN) B LINE | PI655991 | USA |  | Breeding |
| PI655992 | Tx3197 (COMB KAFIR 60) B LINE | PI655992 | USA |  | Breeding |
| PI655993 | Tx399(DALHART RES WHTLND)B LN | PI655993 | USA |  | Breeding |
| PI655994 | WILEY | PI655994 | USA |  | Breeding |
| PI655995 | DEER | PI655995 | USA |  | Cultivar |
| PI655996 | RTx430 | PI655996 | USA |  | Breeding |
| PI655997 | RTx431 | PI655997 | USA |  | Breeding |
| PI655998 | KS19 | PI655998 | USA |  | Breeding |
| PI655999 | Tx2784 | PI655999 | USA |  | Breeding |
| PI656000 | RTx432 | PI656000 | USA |  | Breeding |
| PI656001 | Tx2783 | PI656001 | USA |  | Breeding |
| PI656002 | BOK11 | PI656002 | USA |  | Breeding |
| PI656003 | B AZ9504 | PI656003 | USA |  | Breeding |
| PI656004 | RTx435 | PI656004 | USA |  | Breeding |
| PI656005 | 01MN1589-B | PI656005 | USA |  | Breeding |
| PI656006 | 88V1080 | PI656006 | USA |  | Breeding |
| PI656007 | R9188 | PI656007 |  |  | Cultivar |
| PI656008 | RCV | PI656008 | LAC |  | Cultivar |
| PI656009 | RTAM428 | PI656009 | USA |  | Breeding |
| PI656010 | RTx2536 | PI656010 | USA |  | Breeding |
| PI656011 | 90EON328 | PI656011 | USA |  | Cultivar |
| PI656012 | 90M | PI656012 | USA |  | Breeding |
| PI656013 | 94Q63 | PI656013 | EAS |  | Cultivar |
| PI656014 | Acme Broomcorn | PI656014 |  |  | Breeding |
| PI656015 | Ajabsido | PI656015 | NAF |  | Cultivar |
| PI656016 | B KS66 | PI656016 | USA |  | Breeding |
| PI656017 | 100M | PI656017 | USA |  | Breeding |
| PI656018 | BTx2752 | PI656018 | USA |  | Breeding |
| PI656019 | BTx3042 | PI656019 | USA |  | Breeding |
| PI656020 | BTx406 | PI656020 | USA |  | Breeding |
| PI656021 | 44M | PI656021 |  |  | Breeding |
| PI656022 | BTx615 | PI656022 | USA |  | Breeding |
| PI656023 | Segaolane | PI656023 |  |  | Cultivar |
| PI656023_redo |  | PI656023_redo |  |  | Cultivar |
| PI656024 | SEPON82 | PI656024 |  |  | Cultivar |
| PI656025 | Shan Qui Red | PI656025 | EAS | Bicolor | Cultivar |
| PI656026 | SOBERANO | PI656026 |  |  | Cultivar |
| PI656027 | SRN39 | PI656027 |  |  | Cultivar |
| PI656028 | Town | PI656028 | SAF |  | Cultivar |
| PI656029 | BTx642 | PI656029 | USA |  | Breeding |
| PI656030 |  | PI656030 | USA |  | Breeding |
| PI656031 | CE151-262-A1 | PI656031 |  |  | Cultivar |
| PI656032 | CE260-12-1-1 | PI656032 | WAF |  | Cultivar |
| PI656033 | 60M | PI656033 | USA |  | Breeding |
| PI656034 | Dorado | PI656034 |  |  | Cultivar |
| PI656035 | El Mota | PI656035 | WAF |  | Cultivar |
| PI656036 | ICSV 1089BF | PI656036 |  |  | Cultivar |
| PI656037 | ICSV 401 | PI656037 | SAF |  | Cultivar |
| PI656038 | IS 8525(J) | PI656038 |  |  | Cultivar |
| PI656039 | JOCORO | PI656039 |  |  | Cultivar |
| PI656040 | K886 | PI656040 |  |  | Cultivar |
| PI656041 | 80M | PI656041 | USA |  | Breeding |
| PI656042 | NSA440 - KARPER | PI656042 |  |  | Cultivar |
| PI656043 | KAT83369 | PI656043 |  |  | Cultivar |
| PI656044 | Kuyuma | PI656044 | SAF |  | Cultivar |
| PI656046 | LianTang Ai | PI656046 | EAS | Bicolor | Cultivar |
| PI656047 | M35-1 | PI656047 | SAS |  | Cultivar |
| PI656048 | Malisor 84-7 | PI656048 | WAF |  | Cultivar |
| PI656049 | Marupantse | PI656049 | SAF |  | Cultivar |
| PI656050 | Mota Maradi | PI656050 |  |  | Cultivar |
| PI656051 | MR732 | PI656051 | WAF |  | Cultivar |
| PI656053 | N290B | PI656053 | USA |  | Breeding |
| PI656054 | N.Tenimissa | PI656054 | WAF |  | Cultivar |
| PI656055 | P-721 | PI656055 | USA |  | Cultivar |
| PI656056 | P850029 | PI656056 | USA |  | Breeding |
| PI656057 | P898012 | PI656057 | USA |  | Cultivar |
| PI656058 | P9517 | PI656058 | USA |  | Breeding |
| PI656059 | Pinolero 1 | PI656059 | LAC |  | Cultivar |
| PI656060 | QL3-TEXAS | PI656060 |  |  | Breeding |
| PI656061 | QL3(India) | PI656061 | SAS |  | Cultivar |
| PI656062 | 96CD635 | PI656062 |  |  | Uncertain |
| PI656063 | BQL41 | PI656063 |  |  | Uncertain |
| PI656064 | Chiragon-2 | PI656064 |  |  | Uncertain |
| PI656065 | Koro Kollo | PI656065 |  |  | Cultivar |
| PI656066 | Lakahiri | PI656066 |  |  | Uncertain |
| PI656067 | Rambharose | PI656067 |  |  | Uncertain |
| PI656068 | SC 170-6-17 | PI656068 |  |  | Uncertain |
| PI656069 | SC 326-6 | PI656069 |  |  | Uncertain |
| PI656070 | SC 748-5 | PI656070 |  |  | Uncertain |
| PI656071 | SC 1019 | PI656071 | EAF | Caudatum | Breeding |
| PI656072 | SC 1047 | PI656072 |  |  | Breeding |
| PI656073 | SC 1074 | PI656073 | WAF | Kafir | Breeding |
| PI656074 | SC 1218 | PI656074 | NAF | Guinea | Breeding |
| PI656076 | SC 1271 | PI656076 | EAF | Caudatum | Breeding |
| PI656077 | SC 1416 | PI656077 | WAF | Durra | Breeding |
| PI656078 | SC 1424 | PI656078 | WAF | Kafir | Breeding |
| PI656079 | SC 1426 | PI656079 | WAF | Guinea | Breeding |
| PI656080 | SC 1429 | PI656080 | EAF | Guinea | Breeding |
| PI656081 | SC 1439 | PI656081 |  |  | Breeding |
| PI656082 | SC 145 | PI656082 | EAF | Bicolor | Breeding |
| PI656083 | SC 1451 | PI656083 | EAF | Caudatum | Breeding |
| PI656085 | SC 1465 | PI656085 | NAF |  | Breeding |
| PI656086 | SC 1471 | PI656086 | NAF | Durra | Breeding |
| PI656087 | SC 1476 | PI656087 | NAF |  | Breeding |
| PI656088 | SC 1484 | PI656088 | EAF |  | Breeding |
| PI656089 | SC 1489 | PI656089 | EAF | Durra | Breeding |
| PI656090 | SC 1494 | PI656090 | NAF | Guinea | Breeding |
| PI656091 | SC 22 | PI656091 | EAF | Durra | Breeding |
| PI656092 | SC 25 | PI656092 | EAF | Durra | Breeding |
| PI656093 | SC 295 | PI656093 | WAF | Guinea | Breeding |
| PI656094 | SC 301 | PI656094 | WAF | Guinea | Breeding |
| PI656095 | SC 373 | PI656095 | WAF | Caudatum | Breeding |
| PI656097 | SC 480 | PI656097 |  |  | Breeding |
| PI656098 | SC 49 | PI656098 | NAF | Guinea | Breeding |
| PI656099 | SC 498 | PI656099 | SAS | Durra | Breeding |
| PI656100 | SC 500 | PI656100 | SAS | Durra | Breeding |
| PI656101 | SC 525 | PI656101 | WAF | Guinea | Breeding |
| PI656102 | SC 59 | PI656102 | NAF | Caudatum | Breeding |
| PI656103 | SC 610 | PI656103 | EAS | Bicolor | Breeding |
| PI656104 | SC 621 | PI656104 | SAS | Bicolor | Breeding |
| PI656105 | SC 639 | PI656105 | SAS | Kafir | Breeding |
| PI656106 | SC 695 | PI656106 | EAF | Caudatum | Breeding |
| PI656107 | SC 702 | PI656107 | NAF | Caudatum | Breeding |
| PI656108 | SC 833 | PI656108 | SAS | Durra | Breeding |
| PI656109 | SC 85 | PI656109 | EAF | Caudatum | Breeding |
| PI656110 | SC 968 | PI656110 | SAF | Durra | Breeding |
| PI656111 | SC 971 | PI656111 | USA | Kafir | Breeding |
| PI656112 | SC 1215 | PI656112 | WAF | Guinea | Breeding |
| PI656113 | SC 1277 | PI656113 | SAS | Durra | Breeding |
| PI656114 | SC 134 | PI656114 | EAF | Durra | Breeding |
| PI656115 | SC 1440 | PI656115 | WAF | Guinea | Breeding |
| PI656116 | SC 1506 | PI656116 | WAF |  | Breeding |
| PI656118 | SC 332 | PI656118 | SAS | Durra | Breeding |
| PI656119 | SC 386 | PI656119 | WAF | Caudatum | Breeding |
| PI656120 | SC 790 | PI656120 | SAS | Caudatum | Breeding |
| PI656121 | SC 947 | PI656121 | SAS |  | Breeding |
| PI659691 | COMBINE HEGARI | PI659691 | USA |  | Breeding |
| PI659694 | Tortillerio | PI659694 | SAS |  | Breeding |
| PI659695 | IS 12623C | PI659695 |  |  | Breeding |
| PI659696 | SC 720 | PI659696 | EAF | Caudatum | Breeding |
| PI659753 | IS 5590C | PI659753 |  |  | Breeding |
| PI660555 | Nyahila | PI660555 | EAF | Kafir | Uncertain |
| PI660556 | IS 3627 | PI660556 | WAF | Guinea | Uncertain |
| PI660559 | IS 7419 | PI660559 | WAF | Guinea | Uncertain |
| PI660560 | IS 7427 | PI660560 | WAF | Guinea | Uncertain |
| PI660560_dup | IS 7427_dup | PI660560_dup | WAF | Guinea | Uncertain |
| PI660562 | IS 7920 | PI660562 | WAF | Guinea | Uncertain |
| PI660564 | Bank oumano ziamri fing | PI660564 | WAF | Guinea | Uncertain |
| PI660565 | Kamjin | PI660565 | WAF | Guinea | Uncertain |
| PI660566 | Lekkite bundi | PI660566 | WAF | Guinea | Uncertain |
| PI660571 | Chanan Singoo | PI660571 | SAS | Caudatum | Uncertain |
| PI660580 | Kikuma | PI660580 | EAF | Caudatum | Uncertain |
| PI660582 | Faria bomkum | PI660582 | WAF | Caudatum | Uncertain |
| PI660585 | IS 8087 | PI660585 | EAS | Caudatum | Uncertain |
| PI660587 | Janjari | PI660587 | WAF | Caudatum | Uncertain |
| PI660590 | IS 7254 | PI660590 | WAF | Guinea | Uncertain |
| PI660592 | IS 3911 | PI660592 | SAS | Kafir | Uncertain |
| PI660595 | IS 7435 | PI660595 | WAF | Guinea | Uncertain |
| PI660597 | Kanura maima-saba | PI660597 | WAF | Caudatum | Uncertain |
| PI660601 | Kaura | PI660601 | WAF | Guinea | Uncertain |
| PI660602 | Kaura | PI660602 | WAF | Guinea | Uncertain |
| PI660606 | IS 8100 | PI660606 | EAS | Caudatum | Uncertain |
| PI660608 | Cholia talijhari | PI660608 | SAS | Kafir | Uncertain |
| PI660612 | IS 3814 | PI660612 | SAS | Durra | Uncertain |
| PI660616 | Jowar kalgundi | PI660616 | SAS | Durra | Uncertain |
| PI660618 | Shenoli local | PI660618 | SAS | Durra | Uncertain |
| PI660631 | Line 502 | PI660631 | WAF | Guinea | Uncertain |
| PI660640 | Korky | PI660640 | NAF | Caudatum | Uncertain |
| PI660643 | Gadu umel Tatil | PI660643 | NAF | Caudatum | Uncertain |
| PI660645 | NSL365799 | PI660645 | NAF | Caudatum | Uncertain |
| PI665074 | IS 3614 | PI665074 | WAF | Guinea | Uncertain |
| PI665075 | IS 7429 | PI665075 | WAF | Guinea | Uncertain |
| PI665076 | IS 7550 | PI665076 | WAF | Guinea | Uncertain |
| PI665083 | IS 7779 | PI665083 | WAF | Guinea | Uncertain |
| PI665092 | IS 1207 | PI665092 | EAF | Kafir | Uncertain |
| PI665103 | IS 7380 | PI665103 | WAF | Guinea | Uncertain |
| PI665104 | IS 7738 | PI665104 | WAF | Guinea | Uncertain |
| PI665116 | IS 5554 | PI665116 | SAS | Durra | Uncertain |
| PI665117 | IS 1340 | PI665117 | EAF | Caudatum | Uncertain |
| PI665118 | IS 1144 | PI665118 | SAS | Durra | Uncertain |
| PI665124 | IS 5146 | PI665124 | SAS | Durra | Uncertain |
| PI665129 | IS 5792 | PI665129 | SAS | Durra | Uncertain |
| PI665133 | IS 4870 | PI665133 | SAS | Durra | Uncertain |
| PI665138 | IS 6368 | PI665138 | SAS | Durra | Uncertain |
| PI665144 | IS 6456 | PI665144 | SAS | Caudatum | Uncertain |
| PI665145 | IS 6457 | PI665145 | SAS | Durra | Uncertain |
| PI665146 | IS 5674 | PI665146 | SAS |  | Uncertain |
| PI665147 | IS 6394 | PI665147 | SAS | Durra | Uncertain |
| PI665150 | IS 2198 | PI665150 | SAS | Durra | Uncertain |
| PI665159 | CE 51-69 | PI665159 | WAF | Caudatum | Uncertain |
| PI665160 | Bigheaded Kafir | PI665160 | WAF | Caudatum | Uncertain |
| PI673843 |  | PI673843 | NAF |  | Cultivar |
| PI92270 | IS12748 | PI92270 | EAS | Complex | Cultivar |
| QL12 | QL12 |  | AUS | Complex | Cultivated |
| rio |  |  | USA | Durra | Cultivar |
| rio_dup |  |  | USA | Durra | Cultivar |
| RTx7000 |  |  |  |  | Cultivated |
| S_propinquum_369-1 |  |  |  | Wild | Wild |
| SC103-14E | SC103-14E |  | SAF | Guinea | Landrace |
| SC108C | SC108C |  | EAF | Caudatum | Landrace |
| SC170-6-17 |  |  |  |  | Landrace |
| SC23 | No. 65 Amelsie, Kimbolcha Ethiopia |  | EAF | Durra | Breeding |
| SC237-14E | SC237-14E |  | NAF | Caudatum | Landrace |
| SC326-6 | SC326-6 |  | USA | Caudatum | Landrace |
| SC56-14E | SC56-14E |  | NAF | Caudatum | Landrace |
| SC62C | SC62C |  | EAF | Complex | Landrace |
| Std-Broomcorn |  |  |  |  | Uncertain |
| Tx7000 |  |  | USA | Kafir | Cultivated |
| Yik_solate_IBC_E-339 |  |  | EAF | Durra | Landrace |

Supplemental Table 2.

| Name | Sorghum988 |
| --- | --- |
| Reference Genome | Sbicolor_730_v5 |
| Number of Accessions | 988 |
| Genome Size | 719,894,357 |
| Total Variant Positions | 55,201,900 |
| Total SNP Positions | 47,739,900 |
| Total Indel Positions | 6,674,568 |
| Mean of Missing Data Per Accession | 9,824,315 |
| Number of Genes | 32,160 |
| Number of Genes with Only Reference Allele | 6,375 |
| Number of Genes with Null Alleles | 18,806 |
| Number of Modifying Variants (SNPs and Indels) | 1,901,819 |
| Number of Null Variants (SNPs and Indels) | 13,918 |
| Mean of Number of Variant Positions Per Gene | 29 |
| Median of Number of Variant Positions Per Gene | 21 |
| Mean of Number of Alleles Per Gene | 28 |
| Median of Number of Alleles Per Gene | 18 |

Supplemental Figure S1. The SorghumHub landing page within the KBCommons genomic web portal

SorghumHub serves as the centralized, community focused resource for sorghum genomics and genetics data. The landing page provides organized access to a suite of integrated tools and databases. Key sections directly accessible from this page include the Sorghum Allele Catalog Tool for analyzing genetic variation across the Sbicolor988v5.1 diversity panel and Protein Sequence Logos tool for sequence homology searches. This portal integrates genomic data, genetic variants, and phenotypes, providing a unified starting point for all sorghum-related research. <https://kbcommons.org/system/home/Sbicolor/SorghumHub>

Supplemental Figure S2. Genotype-phenotype association analysis for plant height with the flowering time gene SbFT12 (Sobic.006G047700) in the Sorghum Allele Catalog Tool

As described for Figure 6, the allele catalog data selection interface allows users to filter germplasm based on specific modifying variant positions (allele-defining positions of SbFT12). "Plant Height" was selected to be viewed with position Chr06:36805664 (frameshift variant G171fs). The Violin plot generated by the tool visualizes the phenotype distribution for each genotype for this key agronomic trait, including basic statistics of median (solid line), mean (dashed line), quartiles 1 and 3 (q1 and q3), as well as minimum and maximum. Hovering over each plot allows users to illustrate the value at any position on the plot (x) to compare differences, as in the case here where the x position on the GC reference genotype plot is aligned with the dashed phenotypic mean marking on the G frameshift plot that allows the user to calculate a mean difference of 37 cm taller for accessions with the reference allele. A *t*-test confirmed significance at *p-*value*=*0.013.

Supplemental Figure S3. SorghumHub tool analyses of the betaine aldehyde dehydrogenase (BADH2) gene responsible for fragrance. Partial Sorghum Protein Sequence Logos results for Sobic.007G130800 for amino acid conservation between position 79 and 158. Amino acid position 110 is indicated by orange underline; position 111 is indicated by light blue.
